# Evaluating model robustness in landslide susceptibility mapping using a unified data-consistent framework in northern Thailand

**DOI:** 10.1038/s41598-026-50703-y

**Published:** 2026-05-18

**Authors:** Pichawut Manopkawee, Niti Mankhemthong, Meetaoh Tongsae, Natthaphon Panna

**Affiliations:** Department of Geological Sciences, Faculty of Science, Chiang Mai University, Chiang Mai, 50200 Thailand

**Keywords:** Landslide susceptibility mapping, Machine learning, Deep learning, Luang Prabang Range, Random Forest, Climate sciences, Environmental sciences, Hydrology, Natural hazards

## Abstract

Landslide susceptibility mapping (LSM) is a critical tool for hazard mitigation in mountainous regions. However, the reliability of existing models remains uncertain due to inconsistencies in data quality, sampling strategies, and validation approaches. Although machine learning (ML) and deep learning (DL) models often report high predictive accuracy, their performance may not be robust or transferable, particularly in data-constrained tropical environments. To address this limitation, this study develops a unified, data-consistent evaluation framework to compare statistical, ML, and DL approaches for landslide susceptibility mapping in the Luang Prabang Range, northern Thailand. Five models, including Frequency Ratio (FR), Random Forest (RF), Extreme Gradient Boosting (XGBoost), Convolutional Neural Network (CNN), and Long Short-Term Memory (LSTM), were applied using the same landslide inventory, conditioning factors, and validation strategy to ensure a fair comparison. The results indicate that RF achieved the most stable and reliable performance (AUC = 0.942), followed by XGBoost (0.930), CNN (0.914), LSTM (0.902), and FR (0.865). While DL models demonstrated strong predictive capability, their performance was more sensitive to data limitations and model configuration. In contrast, RF provided a better balance between accuracy, robustness, and data efficiency. The findings demonstrate that differences in model performance are influenced not only by algorithm selection but also by data structure and parameter settings. The main contribution of the study is the implementation of a unified evaluation framework that enables more reliable assessment of model robustness, uncertainty, and interpretability. This study provides practical guidance for model selection in landslide susceptibility mapping and highlights the importance of data consistency and model transparency, particularly in tropical mountainous regions where data limitations are common.

## Introduction

Landslides are among the most destructive natural hazards in mountainous regions, causing significant loss of life, infrastructure damage, and long-term environmental degradation. Their occurrence is controlled by complex interactions among geological, geomorphological, hydrological, and climatic factors, which vary across spatial and temporal scales^1–3^. In tropical mountainous regions, landslide activity is further intensified by the combined effects of heavy rainfall, steep terrain, seismic activity, land-use changes, and uncontrolled development^4–6^. These interacting factors not only increase landslide susceptibility but also complicate the accurate prediction and spatial delineation of hazardous areas. Therefore, reliable landslide susceptibility mapping (LSM) is essential for effective hazard mitigation, land-use planning, and disaster risk reduction^7,8^.

Northern Thailand has experienced significant impacts from landslides and associated flash floods^9^. Although province-scale GIS-based LSMs have been developed in recent years^10^, their effectiveness remains limited due to coarse spatial resolution, outdated datasets, and the lack of integration with dynamic factors, such as localized rainfall and rapid land-use changes. These national-scale maps typically delineate generalized hazard zones based on geology and slope, but fail to provide the spatial precision required for community-level risk management^11,12^. Additional limitations arise from incomplete and biased landslide inventories, particularly in remote mountainous areas where small or shallow failures are often underreported^13^. Furthermore, landslide occurrence in this region is strongly influenced by seasonal monsoonal rainfall, leading to episodic, time-dependent failures that are difficult to capture with conventional static models^14^. The region’s complex terrain, heterogeneous geological conditions, and ongoing land-use changes further obscure the relationships between conditioning factors and landslide occurrence^15^. Collectively, these constraints reduce the predictive capability and generalizability of existing LSM approaches in northern Thailand.

In addition to the map created by governmental agencies^10^, a wide range of methods has been developed for LSM, including heuristic, statistical, and data-driven approaches. Traditional methods such as Frequency Ratio (FR)^16^, Analytic Hierarchy Process (AHP)^17,18^, and Weight of Evidence (WoE)^19^ are widely used due to their simplicity and ease of implementation. However, these approaches rely on simplifying assumptions, including factor independence and linear relationships between conditioning variables and landslide occurrence, which limit their ability to capture the complex, nonlinear nature of landslide processes. In addition, subjective weighting schemes in some methods may introduce bias and reduce model reproducibility and transferability.

To overcome these limitations, machine learning (ML) techniques such as Random Forest (RF) and Extreme Gradient Boosting (XGBoost) have been increasingly applied in LSM. These models can effectively capture nonlinear relationships and interactions among multiple variables, often achieving high predictive performance, with area under the curve (AUC) values exceeding 0.85–0.95 under well-prepared data conditions^20,21^. More recently, deep learning (DL) approaches, including Convolutional Neural Network (CNN) and Long Short-Term Memory (LSTM) networks, have demonstrated strong potential for extracting complex spatial and temporal patterns from large datasets^22^. Despite these advances, both ML and DL models remain highly dependent on input data quality, sampling strategies, and feature selection. Variations in landslide inventory composition, particularly the ratio and spatial distribution of landslide and non-landslide samples, can significantly influence model outputs, leading to inconsistent susceptibility classifications even when the same algorithm is applied^23^. This issue is especially critical in tropical mountainous regions, where incomplete inventories and complex terrain further amplify uncertainty, limiting the robustness and transferability of model predictions^20–22^. Moreover, most studies emphasize predictive accuracy as the primary performance metric while overlooking important aspects of model interpretability and geomorphic relevance^20,21^. Many ML and DL approaches function as “black-box” models, restricting the interpretation of conditioning factor contributions and reducing their applicability in practical hazard management^22,23^.

Despite significant advances in landslide susceptibility mapping, several important limitations remain in current research. Many previous studies have primarily focused on improving predictive accuracy through increasingly complex machine learning and deep learning algorithms, while comparatively less attention has been given to model robustness, uncertainty, and interpretability under consistent data conditions. In addition, comparisons across models are often conducted using different datasets, input variables, parameter settings, and validation strategies, making it difficult to determine whether differences in model performance are due to algorithmic limitations or inconsistencies in data and model configuration^21–23^. These limitations highlight the need for a unified, data-consistent evaluation framework that enables systematic, fair comparison of statistical, machine learning, and deep learning approaches under identical conditions.

Therefore, the novelty of this study lies in the development of a unified modelling framework in which all models are implemented using a common landslide inventory, conditioning factors, and validation procedures. This approach allows a clearer evaluation of model robustness, uncertainty, and interpretability, and reduces bias caused by inconsistent data and parameter settings. Such a framework is particularly important in tropical mountainous regions, where limited data availability and complex environmental conditions can strongly influence model reliability and predictive performance.

The Luang Prabang Range in Nan Province, northern Thailand, represents a critical setting to address these challenges. This mountainous belt, located between the Nan Basin and the Lao PDR, is characterized by complex tectonic structures, intense deformation, and deeply weathered bedrock. The region has experienced repeated landslide and debris flow events, including major events in 2006 and 2018, and the 2025 floods associated with Tropical Storm Wipha^24^. Despite its high susceptibility, systematic high-resolution LSM remains limited, underscoring the need for a robust, data-driven assessment to understand landslide controls better and support regional hazard mitigation. Furthermore, recent studies emphasize the importance of standardized data preparation and multicollinearity control to minimize spatial bias in heterogeneous terrains, aspects that remain insufficiently addressed in current regional frameworks.

To address these challenges, this study systematically evaluates and compares landslide susceptibility models across statistical, machine learning, and deep learning approaches within a unified analytical framework. The Frequency Ratio (FR), Random Forest (RF), Extreme Gradient Boosting (XGBoost), Convolutional Neural Networks (CNN), and Long Short-Term Memory (LSTM) models are applied using the same landslide inventory and conditioning factors in the Luang Prabang Range. By controlling data structure and evaluation conditions, this study quantifies model robustness and uncertainty and assesses whether advanced methods provide reliable improvements over conventional approaches in data-limited environments^20–23^. In addition, the Information Gain Ratio (IGR) is used to evaluate the relative importance of conditioning factors and enhance model interpretability, strengthening the linkage between statistical predictions and underlying geomorphic processes. By explicitly addressing the balance between predictive performance, data dependency, and physical interpretability, this study provides a more reliable basis for model selection and contributes to the development of more robust and transferable landslide susceptibility assessments in tropical mountainous regions.

## The Luang Prabang Range

### Geographical setting of the range

**Fig. 1:**
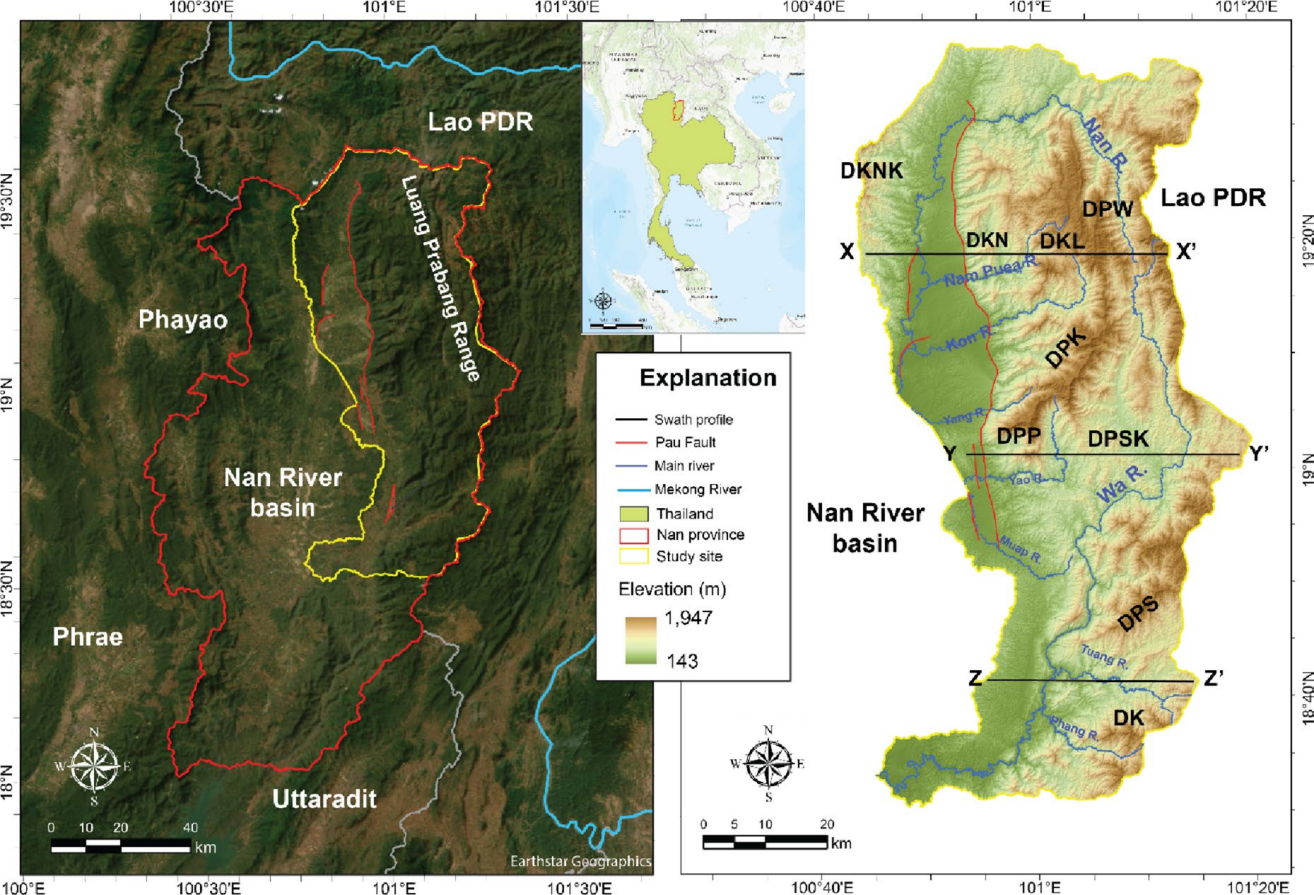
Location of the study area in the Luang Prabang Range (LPR), northern Thailand. The inset shows the location of Nan Province. The LPR is situated between the Nan Basin to the west and Lao PDR to the east. Three topographic swath profiles (X–X′, Y–Y′, and Z–Z′) are indicated. Shapefile data were obtained from the Department of Mineral Resources (DMR), Thailand. The map was generated using ArcGIS Pro version 3.0 (Esri, Redlands, CA, USA; https://www.esri.com), with basemap data from Earthstar Geographics.

The Luang Prabang Range (LPR), a mountain range in the east of northern Thailand, extends from the Mekong River in northeastern Laos to the upper central Thailand. The range is 50–100 km wide and 250 km long, forming part of the Thailand–Lao PDR boundary, and in Thailand, it extends through Nan, Uttaradit, Phitsanulok, Loei, and Phetchabun provinces. In the study, we focus on a section of the mountain range in Nan Province (Fig. 1), which is considered a part of the northern Thai highlands and a major physiographic and tectonic element of mainland Southeast Asia. This mountainous terrain is among the most landslide-prone regions in Thailand, owing to the combined effects of rugged topography, complex geology, intense seasonal rainfall, and strong spatial contrasts in slope gradients, which exert primary control over landform development and hillslope processes.

From a regional geodynamic perspective, the Luang Prabang Range lies between the Sukhothai Terrane and the Indochina Block, in proximity to the Nan–Uttaradit suture zone^25^. The range records the development and subsequent closure of a Nan–Uttaradit back-arc basin associated with the Sukhothai arc, followed by arc–continent collision with the Indochina Block during the Late Paleozoic to Early Mesozoic^26,27^. This tectonic setting has produced a structurally complex mountain belt characterized by intense deformation, metamorphism, faulting, and lithological heterogeneity^28^. Such inherited tectonic complexity exerts a first-order control on slope geometry, rock mass strength, and the spatial distribution of landslide-prone zones.

**Fig. 2:**
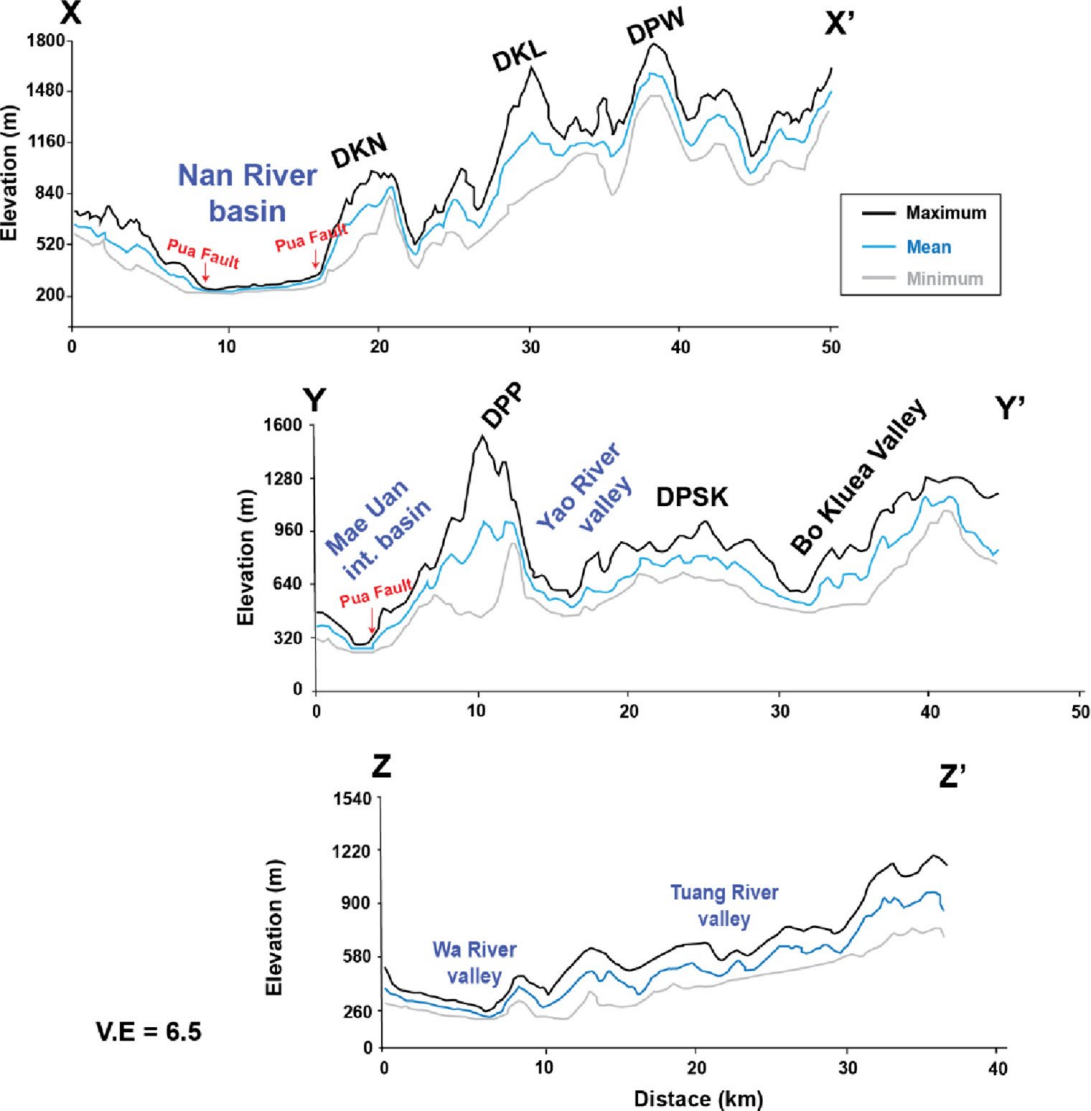
Topographic swath profiles across the Luang Prabang Range: (X–X′) northern profile, (Y–Y′) central profile, and (Z–Z′) southern profile. V.E. denotes vertical exaggeration.

The location and topographic characteristics of the range can be explained by the three primary, parallel swath profiles as X-X’, Y-Y’, and Z-Z’, which cut across low-relief basins in the west and the high mountain blocks in the east (Fig. 1). Along the northern transect (X-X’), the profile represents the Nan River basin bounded by the Pua faults. The valley is located between Doi Khun Nam Kon (DKNK) in the west and Doi Khun Nam Lae (DKN), Doi Khun Nam Leab (DKL), and Doi Phu Wae (DPW) in the east. Elevations in the eastern mountain blocks reach ~ 1,600 m, and the large difference between maximum and minimum elevations indicates rugged terrain and pronounced local relief (Fig. 2), which enhances gravitational driving forces on hillslopes and increases sensitivity to rainfall-triggered failures. The central transect (Y-Y’) crosses the high, undulating mountain blocks of Doi Pha Phueng (DPP) and Doi Phu Sa Kan (DPSK), and the broad intermontane basin of the Mae Uan, the Yao River, and the Bo Kleua valley. The overall topography is high, with the mean elevation generally sustained above 600 m. The lowest point is around 300 m (Fig. 2). The southern transect (Z-Z’) crosses the lower valleys of the Wa River and the small Tuang River. These two valleys are flanked by the peaks of Doi Phu Sanan (DPS) in the north and Doi Khun Lan (DK) in the south, both of which are over 1,300 m. The elevation of the valley floor is typically higher than 500 m (Fig. 2). Across all transects, low-gradient areas along river margins are associated with relatively low erosion rates. In contrast, moderate slopes are widely developed along the foothills of the Luang Prabang Range, where conventional agriculture is limited, and slope-adapted practices are required. The juxtaposition of steep mountain blocks and lower-relief valleys produces strong spatial contrasts in slope angle, drainage density, and sediment accumulation, which are key conditioning factors for landslide susceptibility modeling. In the southern sector, elevated valley floors further indicate limited accommodation space and promote rapid runoff concentration during intense rainfall events.

The physiography of eastern Nan Province is dominated by the mountainous terrain of the LPR, interspersed with hills and lowland plains^29^. Lowland plains with elevations below ~ 300 m occur mainly along the Nan River floodplain within the Nan and Pua basins and are predominantly used for agriculture. These areas transition into uplands, hills, and high plains at elevations of approximately 300–500 m along the western, eastern, and southern margins of the study area. The core of the range consists of moderate- to high-elevation mountains and piedmont slopes, generally between ~ 500 and > 750 m above sea level, with the highest terrain concentrated in the northeastern sector. This elevation zonation reflects strong relief contrasts and exerts a fundamental control on slope gradients, drainage development, and landslide susceptibility across the region.

### Geological setting of the range

**Fig. 3:**
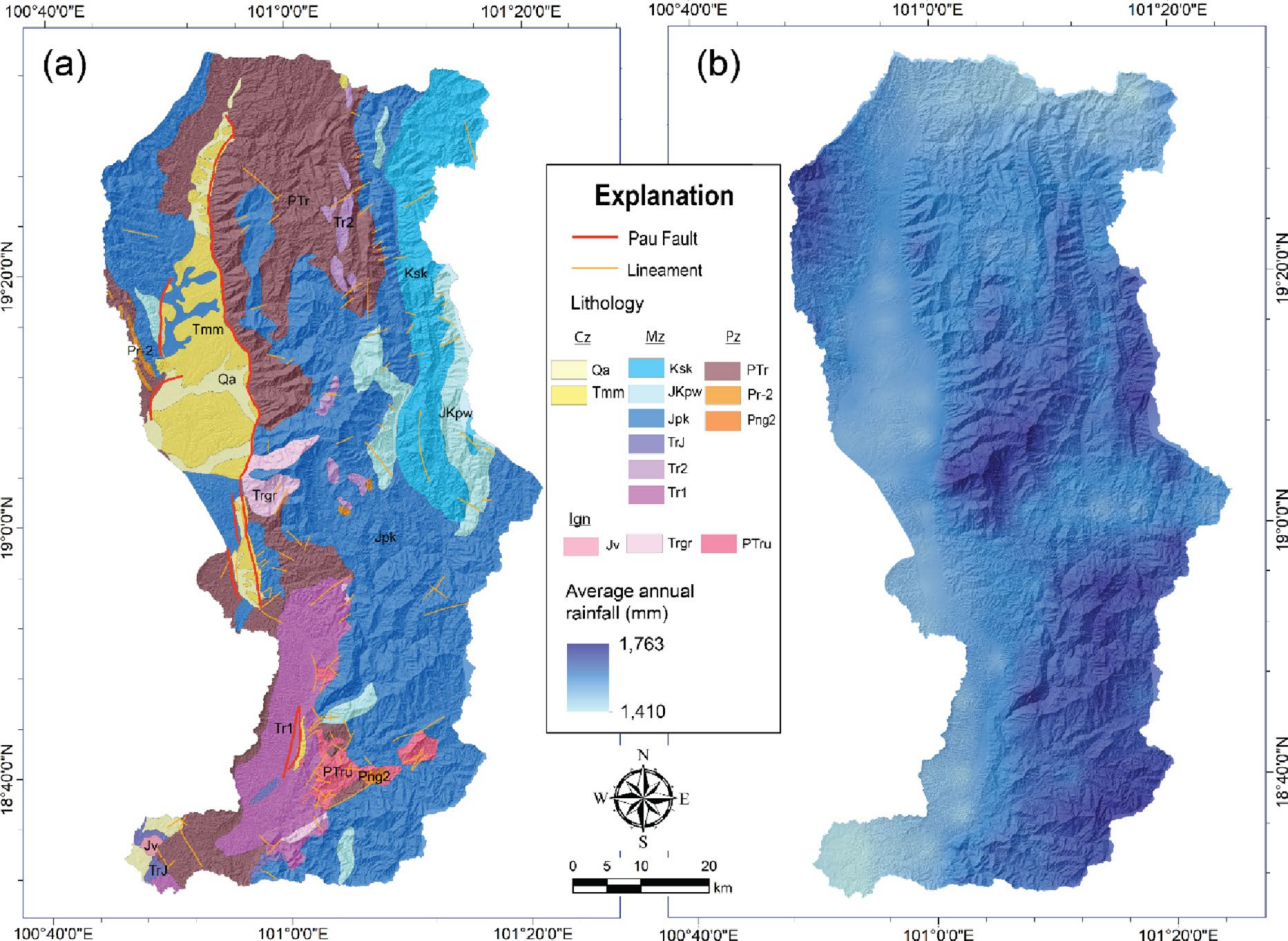
**(a)** Lithological map and **(b)** average annual rainfall distribution of the Luang Prabang Range (LPR). Lithological data were obtained from the Department of Mineral Resources (DMR), Thailand, and rainfall data from the CHIRPS dataset. Maps were generated using ArcGIS Pro version 3.0 (Esri, Redlands, CA, USA; https://www.esri.com).

The basement rocks of the Luang Prabang Range are dominated by Late Paleozoic to Mesozoic sedimentary and metamorphic units, including sandstone, shale, limestone, chert, and locally metamorphosed equivalents, intruded by local-sized granitic bodies^30^. These lithologies are variably fractured and weathered, particularly along major fault zones and shear zones that dissect the range. Permian carbonate rocks are locally.

karstified, while clastic sedimentary units commonly exhibit well-developed bedding and jointing. Both reduce rock mass strength and promote slope instability. The lower basin is covered by large Tertiary alluvial fans and Quaternary alluvial sediment from the Nan River and its tributaries (Fig. 3a).

Evidence of multiple deformation phases is preserved in a network of inherited faults and ductile–brittle structures related to Indosinian orogenesis and later Cenozoic reactivation associated with regional strike-slip tectonics in northern Mainland Southeast Asia, including northern Thailand, Lao PDR, northeastern Myanmar, and southwestern Yunnan (China)^31,32^. N–S-striking normal to oblique dextral-slip Pua faults and related lineament locally control valley orientation, drainage patterns, and slope morphology throughout the mountain range and adjacent intramontane valleys^33^. These structures enhance rock fragmentation and permeability, facilitating deep weathering and groundwater infiltration, which are key preconditioning factors for rainfall-induced landslides.

The range’s climate is strongly influenced by its mountainous topography. It is classified as tropical, corresponding to the tropical savanna climate (Aw) under the Köppen–Geiger climate classification system^34^, and characterized by distinct wet and dry seasons. The rainy season generally extends from May to October, driven primarily by the southwest monsoon, which transports moist air masses from the Indian Ocean and produces prolonged, high-intensity rainfall. During June, the northward migration of the low-pressure trough across southern China may temporarily reduce rainfall, producing a short mid-season dry spell. In addition to the monsoon, tropical storms and typhoons from the South China Sea occasionally affect the region, during the late rainy season (August–October), producing short-duration but extreme rainfall. Total precipitation is highly dependent on elevation due to orographic effects, with higher-altitude areas receiving significantly more precipitation. The average annual rainfall is 1,490 mm, but it may vary significantly within the watershed. It can range from approximately 1,400 mm in the plains to over 1,750 mm in the mountainous regions^35,36^ (Fig. 3b). Such spatial and temporal rainfall variability strongly influences the timing and clustering of landslide events. The dry season lasts from November to April. Temperatures in the range are consistently hot year-round, with average daily temperatures ranging from approximately 21 °C to 35 °C. However, the high-altitude peaks experience a more temperate climate with cooler temperatures^37^. Seasonal contrasts in rainfall and temperature promote cyclic wetting–drying of soils, affecting soil strength, weathering intensity, and slope stability.

**Fig. 4:**
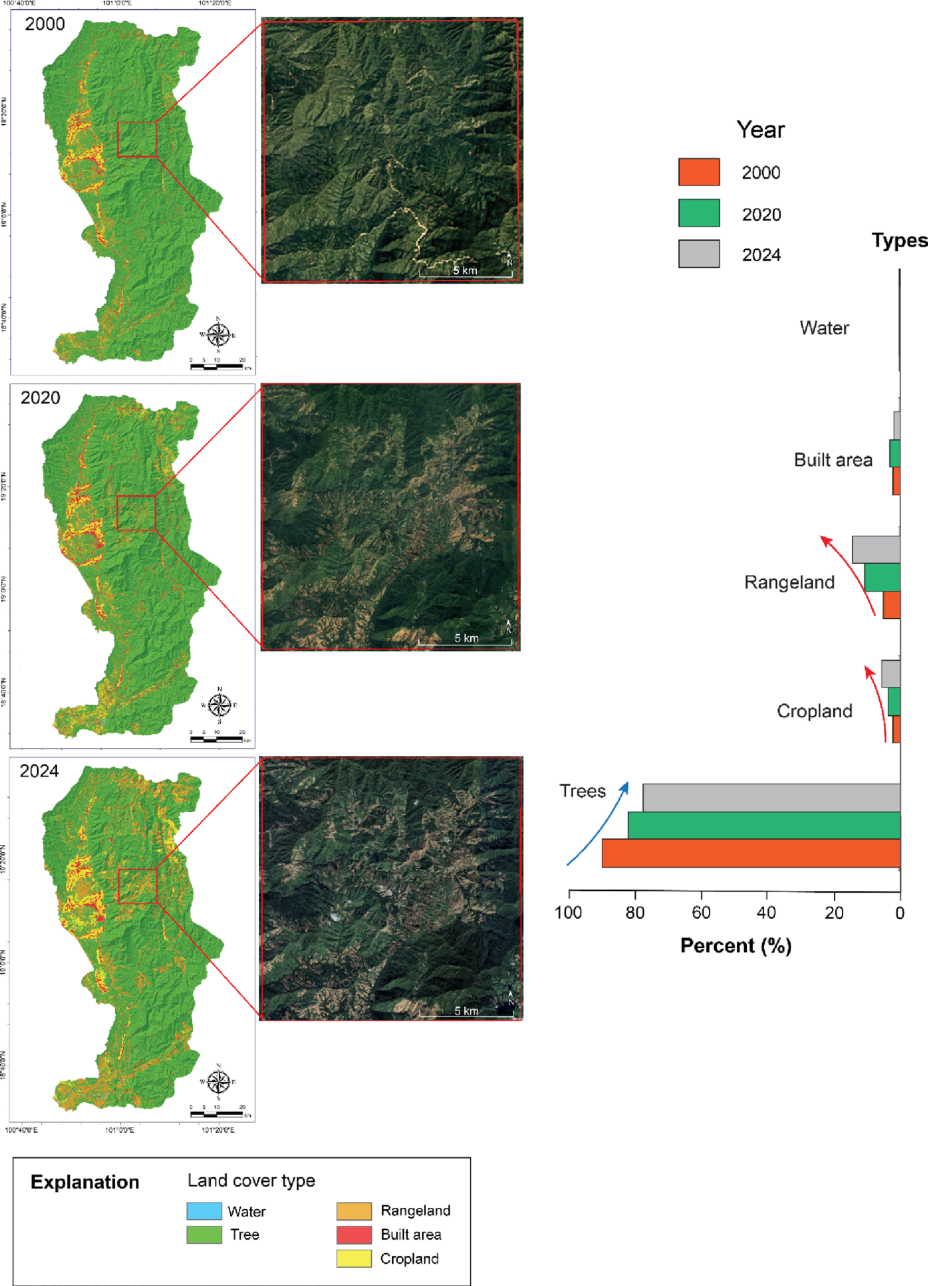
Land use/land cover (LULC) change in the study area. The left panel shows spatial changes for the years 2000, 2020, and 2024, while the right panel presents the percentage change in LULC classes. Data were obtained from the ESA Sentinel-2 dataset (2017–2024).

Superimposed on these climatic controls, recent land-use change has become a major modifier of hillslope processes across the Luang Prabang Range. Over the past few decades, large portions of the mountainous terrain have undergone rapid conversion from natural forests to pasture and corn cultivation^38,39^, particularly on gently undulating to hilly slopes with gradients of approximately 5–35%. Land-cover mapping and vegetation indices indicate a reduction in canopy cover and an increase in seasonal exposure of bare soil in cultivated and abandoned fields^40,41^(Fig. 4). These areas are commonly underlain by shallow to gravelly or lateritic soils that are generally well drained and locally rest on bedrocks, which are frequently encountered within ~ 50 cm of the surface. The combined effects of reduced vegetation cover, limited soil depth, and slope–land use coupling have led to diminished soil moisture retention, weakened root reinforcement, and reduced interparticle cohesion. Consequently, soil erosion rates and shallow landslide occurrence are markedly higher in cultivated and recently abandoned hillslopes than in adjacent forested areas^42^. These anthropogenic disturbances introduce strong spatial heterogeneity and nonlinear geomorphic responses, underscoring the importance of integrating land-use, vegetation, soil, and topographic datasets within data-driven, AI-based frameworks for landslide susceptibility and soil loss assessment.

## Methodology

The key idea of this study is to establish a unified and data-consistent evaluation framework for landslide susceptibility mapping by applying statistical (FR), machine learning (RF, XGBoost), and deep learning (CNN, LSTM) models under identical input conditions. By controlling the landslide inventory, conditioning factors, and validation strategy, this framework enables a fair comparison of model performance, allowing the assessment of robustness, uncertainty, and interpretability rather than relying on predictive accuracy.

**Fig. 5:**
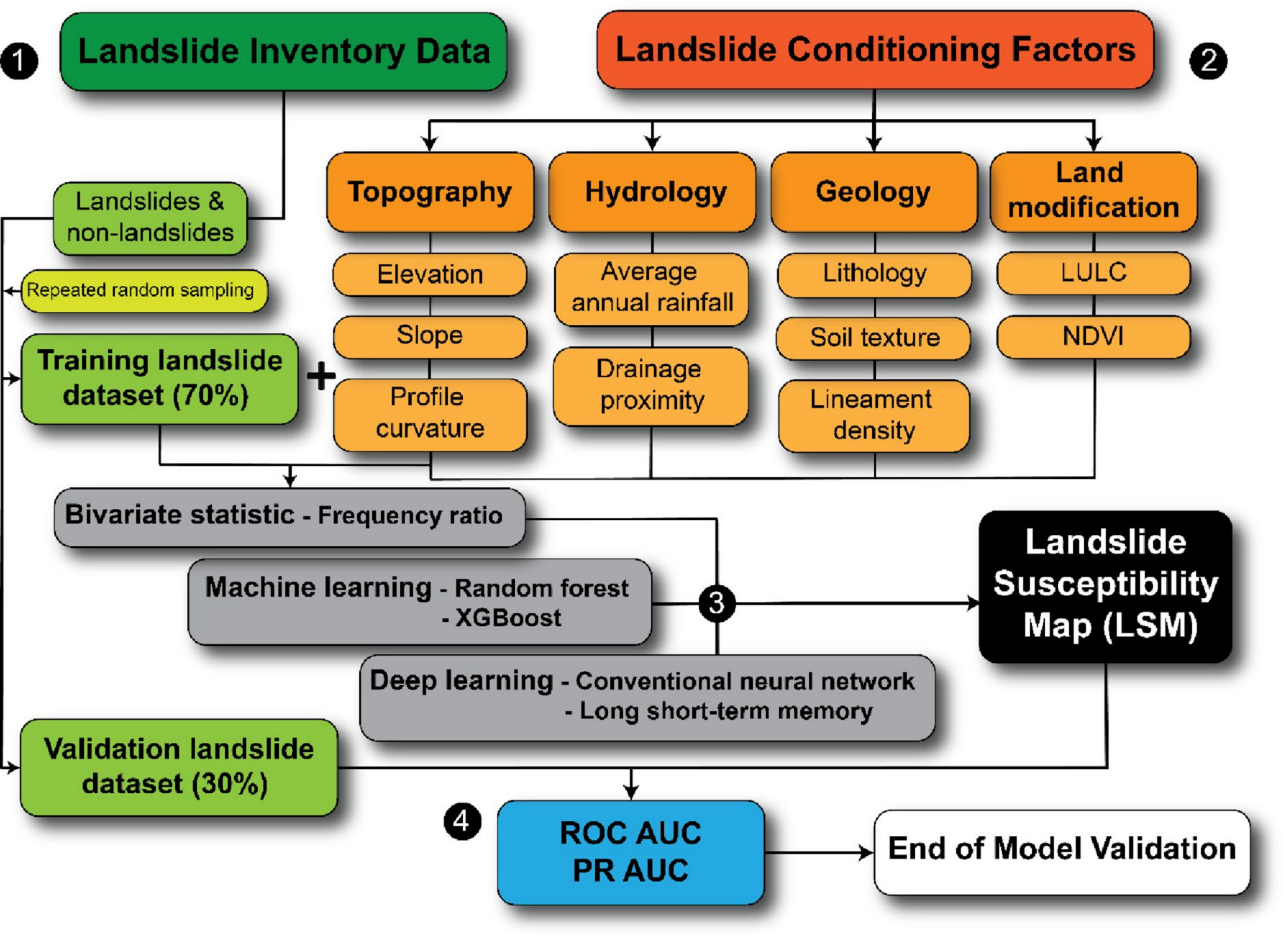
Workflow of landslide susceptibility mapping, including (1) landslide inventory preparation, (2) conditioning factor selection, (3) model development, and (4) model validation.

The workflow consists of four main steps: (1) landslide inventory preparation, (2) selection and preprocessing of landslide conditioning factors, (3) model development via bivariate statistics, ML, and DL methods, and (4) validation and performance evaluation (Fig. 5). This framework enables a systematic comparison of model robustness, uncertainty, and interpretability. All maps and spatial analyses were generated by the authors using ArcGIS Pro version 3.0 (Esri, https://www.esri.com). The datasets used include DEM, CHIRPS rainfall data, Sentinel-2 imagery, and shapefiles from the Department of Mineral Resources, Thailand.

### Landslide inventory and sampling strategy

In this study, careful attention was given to the reliability of landslide and non-landslide samples. As previous research has shown that improper sampling of non-landslide data can introduce significant uncertainty and bias in model performance^43^, this study applied controlled sampling strategies to ensure spatial consistency between landslide and non-landslide datasets.

This study also used the common 1:1 landslide/non-landslide sampling strategy, with repeated random sampling^44,45^. The primary landslide data points were derived from the Department of Mineral Resources (DMR) in Thailand’s landslide scar collection between 1989 and 2020. Additional landslide data were supplemented from historical landslide scars observed in aerial photographs, satellite imagery, and field surveys conducted by DMR’s staff. The non-landslide points were sampled using two combined techniques: (1) randomly sampled outside a 500 m buffer around known landslides and (2) constrained to low-to-moderate susceptibility areas of Thailand to minimize the inclusion of undetected failures, reduce sampling bias, and enhance prediction accuracy^46–48^.

### Dataset partitioning and validation strategy

**Fig. 6:**
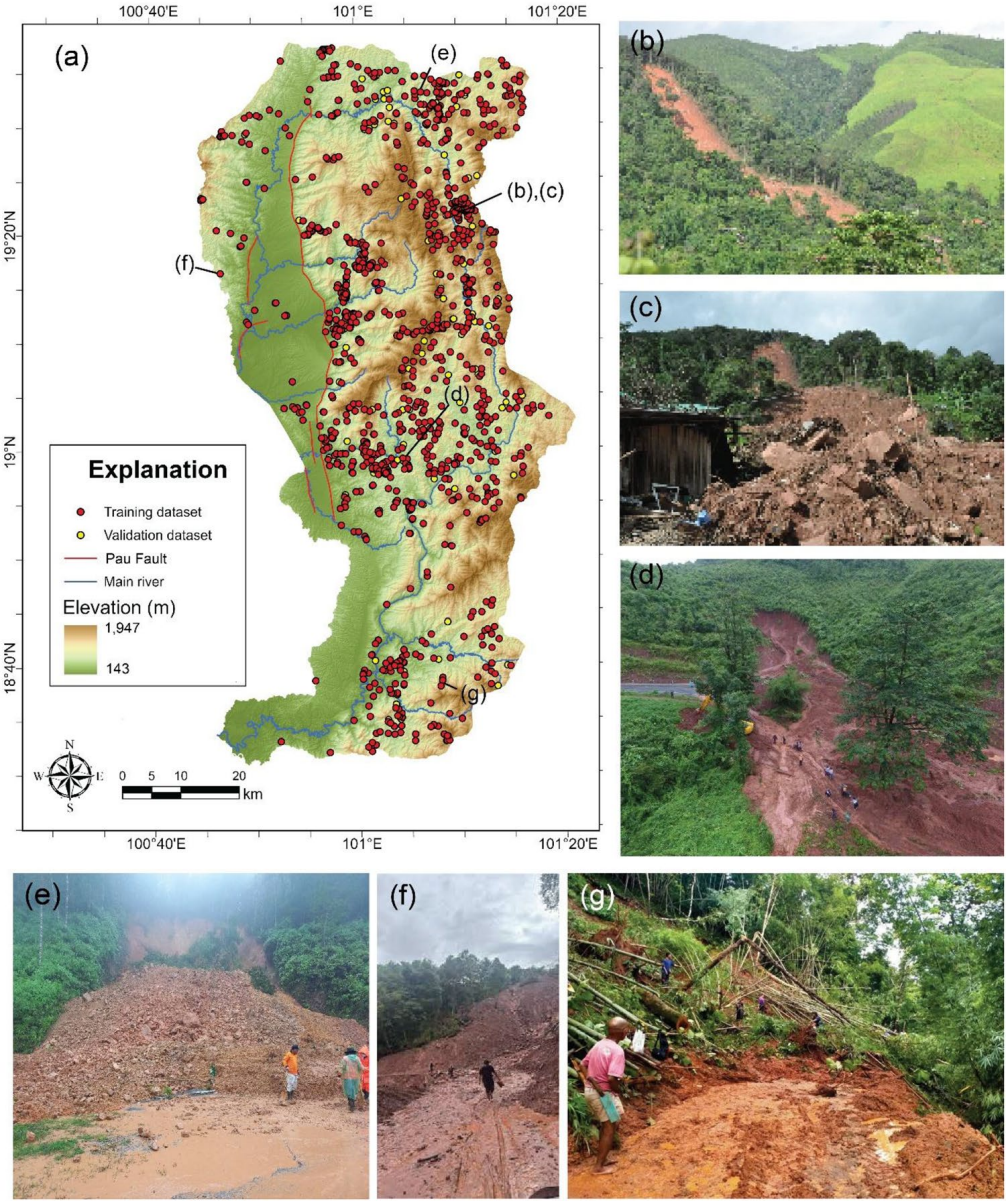
**(a)** Landslide inventory dataset consisting of landslide and non-landslide points generated through repeated random sampling and divided into training (70%) and validation (30%) datasets. **(b–g)** Representative examples of landslide scars observed in the study area. Photographs were captured by the authors and the landslide survey team during field investigations.

The dataset was divided into training (70%) and validation (30%) datasets. The training dataset was used to develop models, while the validation dataset was used to evaluate model performance using validation methods. A buffer-based spatial partitioning approach was applied to separate the training and validation datasets. The approach also ensured that validation samples were located at least 200 m from training data to reduce spatial autocorrelation^49,50^ (Fig. 6a). Model performance was evaluated using receiver operating characteristic (ROC) curves and the area under the curve (AUC), along with additional statistical metrics derived from the confusion matrix. Where available, landslide locations were cross-validated using high-resolution satellite imagery and field observations to verify spatial reliability.

Examples of landslide scars in the range, including the slope collapsed, causing the sliding mass at the toe of the hill of Huay Khab mountain^51^ (Fig. 6b-c), a rainfall-triggered landslide along the Lak Lai-Bo Klua highway in Bo Klua (Fig. 6d), a Thung Chang landslide (Fig. 6e), a Song Kwae landslide (Fig. 6f), and severe flooding and landslides near affected were Ban Huai Lao, Na Noi district^52–54^ (Fig. 6g). Field photographs were collected by the authors and the landslide survey team during site investigations.

### Landslide conditioning factors (LCFs) and data processing

Landslide Conditioning Factors (LCFs) are essential components of landslide susceptibility mapping, as they represent the environmental conditions that influence slope instability. In this study, LCFs were selected based on their relevance to landslide occurrence in Northern Thailand and supported by previous research. Landslides in the region are primarily triggered by prolonged and intense rainfall^9^. Additional factors such as fractured bedrock associated with active faults, steep slopes, high elevation, and land-use changes further contribute to slope failure^55–57^. A total of ten conditioning factors were selected and grouped into four categories: topography, hydrology, geology, and land modification (Table 1).

These factors were derived from multiple data sources and classified into continuous and discrete variables. Continuous factors, including elevation, slope, profile curvature, distance to drainage, and lineament density, were extracted from the 12.5 m ALOS PALSAR digital elevation model (DEM)^58^. Average annual rainfall data were obtained from the Climate Hazards Group InfraRed Precipitation with Station data (CHIRPS) dataset^36^, while vegetation conditions were represented using the Normalized Difference Vegetation Index (NDVI) derived from Sentinel-2 imagery. Discrete factors included lithology, obtained from the Department of Mineral Resources (DMR), and soil texture from the Land Development Department (LDD). Land use/land cover (LULC) data were derived from the European Space Agency (ESA) Sentinel-2 imagery at 10 m resolution dataset (2017–2024). The classification of all conditioning factors was based on expert knowledge and previous studies^16–18^.

All spatial datasets were resampled to a consistent resolution and projected into a common coordinate system to ensure comparability. Prior to model development, correlation analysis was conducted to reduce multicollinearity among variables. In addition, the Information Gain Ratio (IGR) was applied to quantify the relative importance of each conditioning factor, thereby enhancing model interpretability and supporting a better understanding of landslide controls.

#### Topographic-related factors

**Fig. 7:**
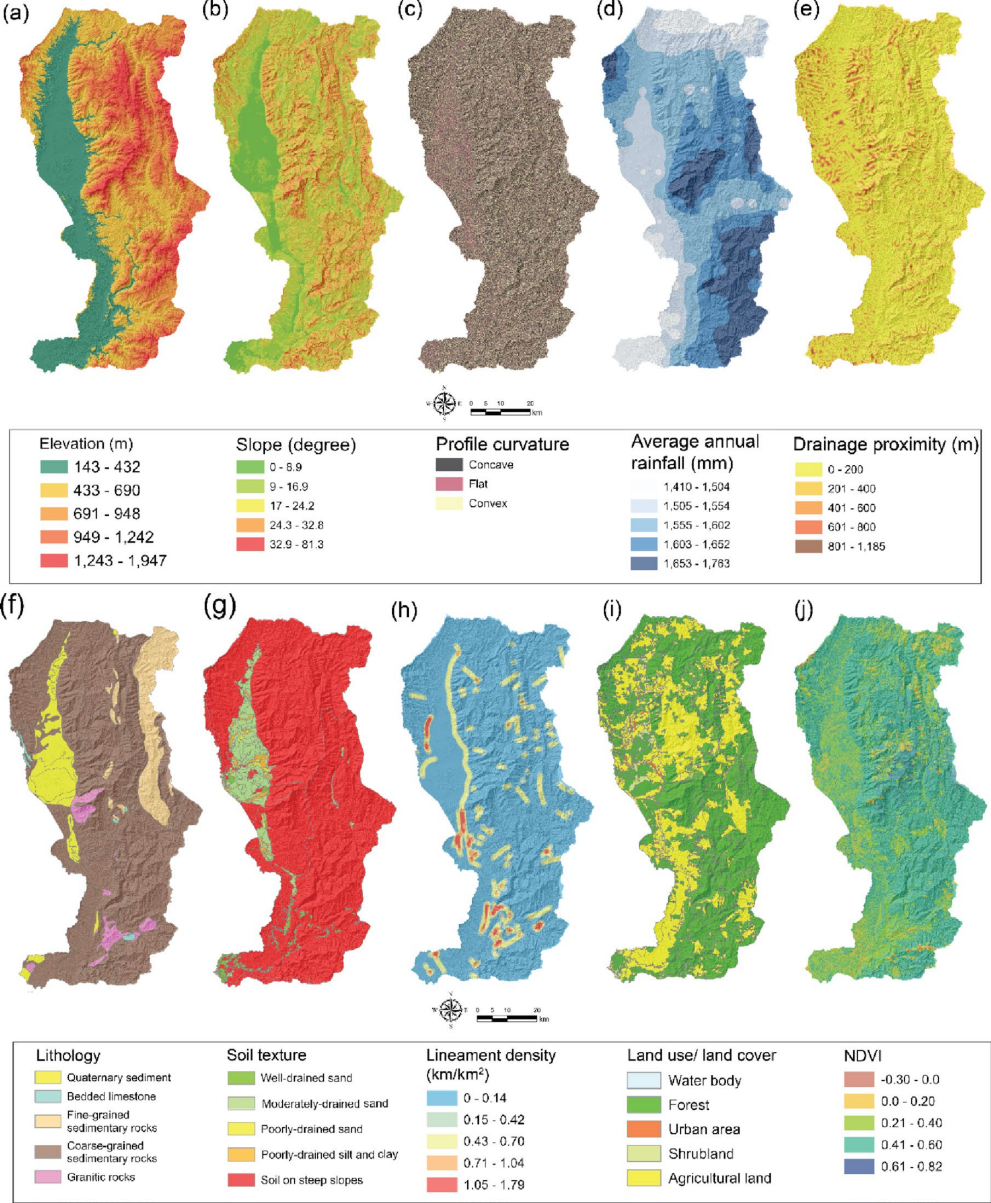
Landslide conditioning factors (LCFs): **(a)** elevation, **(b)** slope, **(c)** profile curvature, **(d)** average annual rainfall, **(e)** drainage proximity, **(f)** lithology, **(g)** soil texture, **(h)** lineament density, **(i)** land use/land cover, and **(j)** Normalized Difference Vegetation Index (NDVI). Elevation-derived factors (a–c, e, h) were extracted from the ALOS PALSAR digital elevation model (DEM). Rainfall data (d) were obtained from the CHIRPS dataset. NDVI (j) and land use/land cover (i) were derived from Sentinel-2 imagery. Lithological data (f) and soil texture (g) were obtained from the Department of Mineral Resources (DMR) and the Land Development Department (LDD), Thailand, respectively. All spatial datasets were processed and maps were generated using ArcGIS Pro version 3.0 (Esri, Redlands, CA, USA; https://www.esri.com).

Elevation is a significant factor influencing the occurrence of landslides. Variations in elevation directly affect slope stability by increasing weathering and altering vegetation types and growth^59,60^. The elevation map was derived from the high-resolution (12.5-meter) digital elevation model (DEM) and classified into five classes using the natural break classification method: 143–432, 433–690, 691–948, 949-1,242, and 1,243-1,947 m (Fig. 7a).

**Table 1:** Data sources used for assessing landslide susceptibility in the Luang Prabang Range (LPR).

Data	Categories	Classification Scheme	Data Sources (year of data used)	Scale/Resolution
Landslide inventory	-	-	DMR (1989–2020)	-
	Topography	Elevation	Topographic Map, DMR (2000) DEM from ALOS PALSAR (2011)	1:50,000 12.5 × 12.5 m
		Terrain slope	DEM from ALOS PALSAR (2011)	12.5 × 12.5 m
		Profile curvature	DEM from ALOS PALSAR (2011)	12.5 × 12.5 m
	Hydrology	Average annual rainfall	CHIRPS (1981–2020)	12.5 × 12.5 m
		Drainage proximity	DEM from ALOS PALSAR (2011)	12.5 × 12.5 m
	Geology	Lithology	Thailand geologic map, DMR (1995)	1:250,000
		Soil texture	Soil series, LDD (2024)	1:25,000
		Lineament density	Thailand geologic map, DMR (1995) DEM from ALOS PALSAR (2011) Landsat 8 OLI/TIRS (2024) Sentinel-2 (2024)	1:250,000 12.5 × 12.5 m 30 × 30 m 10 × 10 m
	Land modification	Land use and land cover (LULC)	ESA LULC maps (2017–2024)	1:50,000 10 × 10 m
		Normalized difference vegetation index (NDVI)	Sentinel-2 vegetation index image series (2025)	10 × 10 m

The slope angle is a key factor influencing slope stability because steeper slopes tend to be less stable, and consequently, lower stability correlates with increased susceptibility to landslides. Numerous studies identified slope angles as a key factor in assessing landslide susceptibility^61,62^. The slope angle map was generated from the DEM in ArcGIS software and categorized into five classes: 0–8.9.9 °, 9–16.9.9 ^o^, 17–24.2.2 ^o^, 24.3–32.8 ^o^, and 32.9–81.3 ^o^ (Fig. 7b).

Profile curvature refers to the curvature in the direction of decline, measured at the intersection of a vertical plane and the terrain surface^63,64^. Concave slopes (indicating negative curvature) are more prone to landslides because they gather surface water and debris, which raises pore water pressure and diminishes soil strength. On the other hand, convex slopes (with positive curvature) divert channel water, reducing the likelihood of landslides. In contrast, flat surfaces may show an increased risk of landslides, particularly in regions with debris and clay-rich soils. Profile curvatures were obtained from the DEM using SAGA-GIS software and classified into three categories: concave, flat, and convex (Fig. 7c).

#### Hydrological-related factors

Rainfall is a crucial conditioning factor in the initiation and occurrence of landslides^65,66^. Areas in the mountainous terrain with higher cumulative rainfall are prone to landslides. The amount, intensity, and duration of precipitation are three significant factors in evaluating landslide susceptibility because rainfall significantly affects soil moisture, reduces slope stability, and increases the likelihood of landslides^67^. The average annual rainfall collected since 1981, derived from CHIRPS and USGS, was categorized in ArcGIS software into five different classes: 1,410-1,504, 1,505-1,554, 1,555-1,602, 1,603-1,652, and 1,653-1,763 (Fig. 7d).

A river, along with its tributaries, can lead to the erosion of streambanks and the washing away of the base of sloped hills. The stability of these slopes is inversely related to their proximity to the drainage system: the closer the slope is, the greater the basal erosion and the greater the saturation of the hillside material^68^. The drainage network was generated using a built-in script in the ArcGIS hydrology toolset. A map showing proximity to the drainage system was then created by buffering the network at 200-meter intervals. The spatial variation was classified into five categories: 0–200 m, 201–4000 m, 401–600 m, 601–800 m, and 801-1,185 m (Fig. 7e).

#### Geological-related factors

Lithology refers to the type of rock that describes the physical properties of rocks found in a specific area, such as composition, texture, grain size, rock strength, porosity, and permeability. Thus, landslide occurrences and development depend on the quality of rock mass^69,70^. Different lithological units exhibit varying levels of landslide susceptibility across different slope conditions^71^. The lithology map, derived from a DMR geological map, was converted to a raster format in ArcGIS software. The lithological classification of the study area comprised five distinct groups: Quaternary sediments, bedded limestone, fine-grained sedimentary rocks, coarse-grained sedimentary rocks, and granitic rocks (Fig. 7f).

Soil is a product of the disintegration process of rock, which consists of various proportions of gravel, sand, silt, clay particles, and organic materials. The different components of soil reflect the variations in strength, moisture content, and viscosity, which play a crucial role in influencing slope stability^72^. The characteristics and attributes of the soil series within the range were collected by the Land Development Department^73^ and converted to raster format using ArcGIS software. Soil texture was classified into five categories based on location and drainage characteristics: well-drained sand, moderately drained sand, poorly drained sand, poorly drained silt and clay, and soil on steep slopes (Fig. 7g).

The active Pua fault zones bound the LPR to the west^74^. Rock exposure next to these faults has led to the formation of lineaments, such as joints and cracks, which are observed at the surface^75,76^. These lineaments cause slope instability and possible failure by weakening the rock mass and creating channels for water infiltration. Geological structure data from Thailand’s DMR database were combined with finer lineaments derived from image-filtering methods and channel-flow analysis of satellite images using PCI Geomatica^77,78^. Lineament density was calculated using the equation provided by^79^. Variation in lineament density was categorized into five classes based on the natural break classification method: 0–0.14, 0.15–0.42, 0.43–0.70, 0.71–1.04, and 1.05–1.79 km/km^2^ (Fig. 7h).

#### Land modification-related factors

Land use refers to the conversion of natural landscapes into built environments or semi-natural habitats, while land cover is the physical material at Earth’s land surface^80,81^. Changes in land use/land cover result in the loss of vegetation and soil in the region, which can impact slope stability and increase the susceptibility of landslides^82,83^, since landslides tend to be more critical in areas with diminished vegetation. The land use/land cover map was derived from the ESA Sentinel-2 10 m resolution Land Use/Land Cover collected between 2017 and 2024. The map was categorized into five types: water bodies, forests, urban areas, shrublands, and agricultural land (Fig. 7i).

The health and vitality of the vegetation within the watershed can be assessed using the Normalized Difference Vegetation Index (NDVI). NDVI likely indicates landslides by focusing on vegetation changes that trigger ground-surface disturbances, such as the transition from vegetated to bare ground, which corresponds to lower NDVI values. The derivation of NDVI was determined for each pixel in a Sentinel-2 satellite image as a normalized ratio of the difference between the near-infrared (NIR) wavelength (band 8) and the red (RED) wavelength (band 4)^84,85^. The spatial distribution of NDVI values was divided into five categories: <0 (bare soil), 0–0.2.2 (sparse or low vegetation), 0.2–0.4 (moderately low vegetation), 0.4–0.6 (moderately high vegetation), and 0.6–0.82 (dense or high vegetation) (Fig. 7j).

#### Multicollinearity test

To ensure optimal model performance and efficiency, multicollinearity analysis is a crucial step in identifying and selecting appropriate LCFs^86^. In this study, multicollinearity was evaluated using several methods to ensure consistent feature selection, including (1) the Pearson Correlation Coefficient (PCC), (2) Tolerance (TOL) and Variance Inflation Factor (VIF), and (3) Information Gain Ratio (IGR). PCC quantifies the linear relationship between two factors that help identify highly correlated elements that might.

have an impact on model performance^87^. PCC is determined using Eq. (1):

1 $$\:{\mathrm{r}}_{\mathrm{x}\mathrm{y}}=\frac{\sum\:_{\mathrm{i}=1}^{\mathrm{n}}({\mathrm{X}}_{\mathrm{i}}-\mathrm{X})({\mathrm{Y}}_{\mathrm{i}}-\mathrm{Y})}{\sqrt{\sum\:_{\mathrm{i}=1}^{\mathrm{n}}{({\mathrm{X}}_{\mathrm{i}}-\stackrel{-}{\mathrm{X}})}^{2}\sum\:_{\mathrm{i}=1}^{\mathrm{n}}{({\mathrm{X}}_{\mathrm{i}}-\stackrel{-}{\mathrm{Y}})}^{2}}}$$

where $\:\mathrm{n}$ is the sample size, $\:{\mathrm{X}}_{\mathrm{i}}$ and $\:{\mathrm{Y}}_{\mathrm{i}}$ are individual values, and $\:\stackrel{-}{\mathrm{X}}$ and $\:\stackrel{-}{\mathrm{Y}}$ are mean values. The correlation between two variables can range from − 1 to + 1, indicating perfect negative and positive correlation. PCCs of |r| > 0.7 or r^2^ > 0.49 were used to detect significant multicollinearity, which was considered indicative of a strong correlation that destabilizes model predictive efficiency. Therefore, one factor should be removed to ensure the remaining factors remain relatively independent^88,89^.

The degree of multicollinearity between factors was measured using VIF and TOL, where VIF indicates the extent to which other variables in the model explain a given variable^90,91^. The VIF is computed as:

2 $$\:\mathrm{V}\mathrm{I}\mathrm{F}=\:\frac{1}{1-{\mathrm{r}}^{2}}$$

where $\:{\mathrm{r}}^{2}$ is the coefficient of determination. In contrast, TOL provides an inverse indicator of the variance inflation caused by multicollinearity that is computed as:

3 $$\:\mathrm{T}\mathrm{O}\mathrm{L}=\:\frac{1}{\mathrm{V}\mathrm{I}\mathrm{F}}$$

If a VIF value > 10 and a TOL value > 0.10, it is typically considered to indicate significant multicollinearity. A predictor variable explained by the other predictors. The factor should be removed to reduce multicollinearity^92^.

To further improve the feature selection procedure, the IGR was used to measure each factor’s contribution to the overall landslide susceptibility model. IGR is calculated as:

4 $$\:\mathrm{G}\mathrm{R}=\:\frac{\mathrm{G}\mathrm{a}\mathrm{i}\mathrm{n}\:\left(\mathrm{A}\right)}{{\mathrm{S}\mathrm{p}\mathrm{l}\mathrm{i}\mathrm{t}}_{\mathrm{i}\mathrm{n}\mathrm{f}\mathrm{o}}\left(\mathrm{A}\right)}$$

where $\:\mathrm{G}\mathrm{a}\mathrm{i}\mathrm{n}\:\left(\mathrm{A}\right)$ is the expected reduction in entropy from splitting on factor A, and $\:{\mathrm{S}\mathrm{p}\mathrm{l}\mathrm{i}\mathrm{t}}_{\mathrm{i}\mathrm{n}\mathrm{f}\mathrm{o}}\left(\mathrm{A}\right)$ measures the intrinsic information of the split^93,94^. The IGR is especially effective in identifying factors that significantly affect the likelihood of landslides, increasing the model’s precision and effectiveness. The higher the IGR value, the greater the factor’s influence on landslide occurrence. If the IGR value equals 0, the conditioning factor does not influence landslide occurrence^95,96^.

### Landslide susceptibility models (LSMs)

In this study, landslide susceptibility mapping was performed by different methodological domains: the Frequency Ratio (FR) as a statistical method; Random Forest (RF) and Extreme Gradient Boosting (XGBoost) as ML models; and Convolutional Neural Network (CNN) and Long Short-Term Memory (LSTM) as DL approaches.

Following the multicollinearity analysis, 10 selected landslide conditioning factors were used to identify and remove redundant conditioning factors across all models. The FR method was used to evaluate the statistical relationship between conditioning factors and landslide occurrence, while ML and DL models were developed in Python (version 3.12.5) on Google Colab, with data preprocessing using Pandas and NumPy.

To ensure a fair and consistent comparison, all models were trained using the same landslide inventory and conditioning factors, with similar parameter configurations. Individual models were applied rather than ensemble-based approaches to maintain methodological consistency and to allow a controlled evaluation of model performance across statistical, ML, and DL domains.

#### Frequency ratio (FR)

The FR model was selected to evaluate the likelihood of landslides in the range by examining the correlation between landslide distribution and the conditioning factors contributing to them. The model calculates the proportion of the total area under investigation that has experienced landslides^97,98^. Landslide inventory data were split into 70% for training and 30% for validation^99^. This selection was performed at random using the Subset Feature in the Geospatial Analyst tool in ArcGIS software. The formulation of the FR model is presented in Eq. (5):

5 $$\:{\mathrm{F}\mathrm{R}}_{\mathrm{i}\mathrm{j}}=\:\frac{{\mathrm{N}}_{\mathrm{p}\mathrm{i}\mathrm{x}}\left({\mathrm{L}\mathrm{X}}_{\mathrm{i}}\right)/\sum\:_{\mathrm{i}=1}^{\mathrm{m}}{\mathrm{N}}_{\mathrm{p}\mathrm{i}\mathrm{x}}\left({\mathrm{L}\mathrm{X}}_{\mathrm{i}}\right)}{{\mathrm{N}}_{\mathrm{p}\mathrm{i}\mathrm{x}}\left({\mathrm{X}}_{\mathrm{j}}\right)/\sum\:_{\mathrm{j}=1}^{\mathrm{n}}{\mathrm{N}}_{\mathrm{p}\mathrm{i}\mathrm{x}}\left({\mathrm{L}\mathrm{X}}_{\mathrm{j}}\right)}$$

where $\:{\mathrm{N}}_{\mathrm{p}\mathrm{i}\mathrm{x}}\left({\mathrm{L}\mathrm{X}}_{\mathrm{i}}\right)\:$is the number of pixels with landslides within the class $\:\mathrm{i}$ of factor $\:\mathrm{X}$, and $\:{\mathrm{N}}_{\mathrm{p}\mathrm{i}\mathrm{x}}\left({\mathrm{X}}_{\mathrm{j}}\right)\:$is the number of pixels within the factor $\:{\mathrm{X}}_{\mathrm{j}}$, $\:\mathrm{m}$ is the number of classes in the factor $\:{\mathrm{X}}_{\mathrm{i}}$, and $\:\mathrm{n}$ is the number of parameters in the study area^100^. The Tabulate Area function in ArcGIS software was used to intersect the landslide factors with the landslide training dataset, enabling the identification of the number of landslide pixels associated with each factor’s category. The resulting values were computed as the FR values from Eq. (6). To generate the landslide susceptibility index (LSI), all FR values were aggregated using the Raster Calculator in ArcGIS software.

6 $$\:\mathrm{L}\mathrm{S}\mathrm{I}={\mathrm{F}\mathrm{R}}_{1}+{\mathrm{F}\mathrm{R}}_{2}+\dots\:+\:{\mathrm{F}\mathrm{R}}_{\mathrm{n}}$$

where FR is the FR value of each landslide conditioning factor, and 1, 2, 3, and n are the number of conditioning factors.

#### Machine learning (ML) approaches

**Fig. 8:**
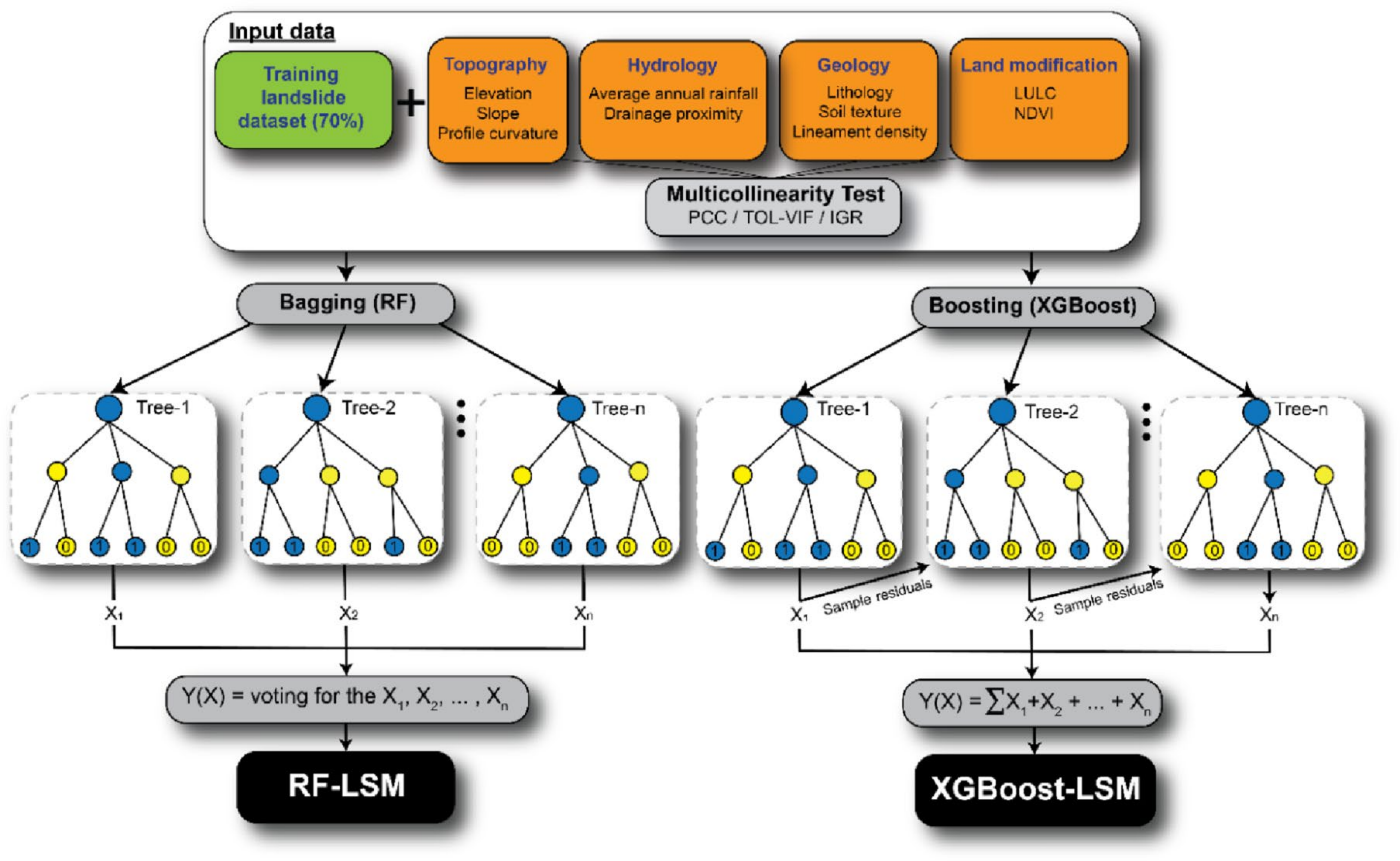
Workflow of the machine learning models used for landslide susceptibility mapping, including Random Forest (RF) and Extreme Gradient Boosting (XGBoost).

Random Forest (RF), developed by^101^, is a machine learning classification method that uses multiple decision trees during training and selects the class with the major vote across the individual trees’ classifications or regressions^102^. RF uses bootstrap aggregating (bagging) to generate multiple distinct training subsets from the original dataset via bootstrap sampling, and then constructs a decision tree for each subset^103,104^. The RF model randomly chooses a subset of features rather than selecting the best splitting point from all available features. Each decision tree is developed using different features and sample subsets, resulting in variability across them. Each decision tree in the RF independently makes judgments and predictions based on the input samples. Ultimately, the predictions from all decision trees are combined using majority voting to produce the final prediction (Fig. 8). The voting process can be explained as follows:

7 $$\:\mathrm{Y}\left(\mathrm{x}\right)={\mathrm{a}\mathrm{r}\mathrm{g}}_{\mathrm{z}}^{\mathrm{m}\mathrm{a}\mathrm{x}}\sum\:_{\mathrm{i}=1}^{\mathrm{k}}\mathrm{I}({\mathrm{y}}_{\mathrm{i}}\left(\mathrm{x}\right)=\mathrm{Z})$$

where Y(x) is the RF model, $\:{\mathrm{X}}_{\mathrm{i}}$ is an individual tree structure, $\:\mathrm{I}$ is the indicative function, and $\:\mathrm{Z}$ is the final prediction. The RF algorithm calculates the significance score for each variable using the Gini index, a metric that quantifies a variable’s impurity with respect to the classes^105^. The RF generally achieves more accurate and reliable predictions than other statistical methods because (1) RF effectively reduces the risk of overfitting, thereby improving the model’s ability to generalize. (2) RF shows a significant tolerance for outliers and noise, remaining largely unaffected by individual trees. (3) Due to the combination of bagging techniques and feature selection, RF demonstrates strong adaptability to various data sets. (4) RF trains relatively quickly, making it suitable for large-scale datasets^106^.

Extreme Gradient Boosting (XGBoost), similar to RF, is an ensemble ML method that combines multiple decision trees as base learners^108^. The key distinction between RF and XGBoost lies in the construction methods. As the RF builds decision trees independently and simultaneously by averaging their votes, XGBoost constructs and evaluates trees iteratively, with each tree dependent on the others. The residual from previous trees is used as input for subsequent trees. XGBoost focuses on learning from misclassified observations by assigning higher weights to subsequent trees^107^. A higher weight signifies a greater influence of a particular class or instance on the loss function. The prediction result is determined by the sum of all trees’ results^108^(Fig. 8). XGBoost can be expressed as:

8 $$\:{\widehat{\mathrm{y}}}_{\mathrm{i}}^{\left(\mathrm{t}\right)}=\:\sum\:_{\mathrm{k}=1}^{\mathrm{t}}{\mathrm{f}}_{\mathrm{k}}\left({\mathrm{x}}_{\mathrm{i}}\right)={\widehat{\mathrm{y}}}_{\mathrm{i}}^{(\mathrm{t}-1)}+{\mathrm{f}}_{\mathrm{t}}\left({\mathrm{x}}_{\mathrm{i}}\right)\:$$

Where $\:{\widehat{\mathrm{y}}}_{\mathrm{i}}^{\left(\mathrm{t}\right)}$is the final predicted possibility of landslide susceptibility, $\:{\widehat{\mathrm{y}}}_{\mathrm{i}}^{(\mathrm{t}-1)}$is the accumulated landslide susceptible probability from previous iterations, $\:{\mathrm{x}}_{\mathrm{i}}$ represents the independent factor, $\:{\mathrm{f}}_{\mathrm{k}}\left({\mathrm{x}}_{\mathrm{i}}\right)\:$represents the newly generated tree in the current iteration to the landslide susceptible probability, and $\:\mathrm{t}$ is the number of model iterations^109^. The loss function of the XGBoost is expressed as:

9 $$\:\mathrm{L}\left({\varnothing}\right)=\:\sum\:_{\mathrm{i}=1}^{\mathrm{n}}\mathrm{l}(\left({\widehat{\mathrm{y}}}^{\mathrm{t}},{\mathrm{y}}^{\mathrm{t}})+{\sum\:}_{\mathrm{k}}{\Omega\:}\left(\mathrm{f}\right)\:\right)$$

10 $$\:{\Omega\:}\left(\mathrm{f}\right)=\:{\upgamma\:}\cdot\:\mathrm{T}+\frac{{\uplambda\:}\cdot\:{\Vert\:{\upomega\:}\Vert\:}^{2}}{2}$$

where $\:\mathrm{L}$ is a loss function, l(_) is the loss function for a single sample, $\:{\mathrm{y}}^{\mathrm{t}}\:$is the true label value of the training sample, $\:{\Omega\:}\left(\mathrm{f}\right)$ is a penalty value representing the complexity of the model, $\:{\upgamma\:}$ and $\:{\uplambda\:}$ are manually set parameters, $\:{\upomega\:}$ is a vector formed by the values of all leaf nodes in the decision tree, and $\:\mathrm{T}$ is the number of leaf nodes^110^.

#### Deep learning (DL) approaches

Convolutional Neural Network (CNN) is a DL method that has become an effective approach for landslide susceptibility mapping (LSM) because it can automatically learn complex spatial features from multi-source geospatial data. Unlike traditional qualitative or statistical methods that rely on manual feature selection, CNN directly extracts nonlinear patterns from landslide conditioning factors. CNN requires conditioning factors prepared as multi-channel raster layers and organized into landslide and non-landslide samples, typically using grid-based image patches. This structure enables the CNN to capture both local values and surrounding spatial context, which is critical for representing slope processes^111,112^.

**Fig. 9:**
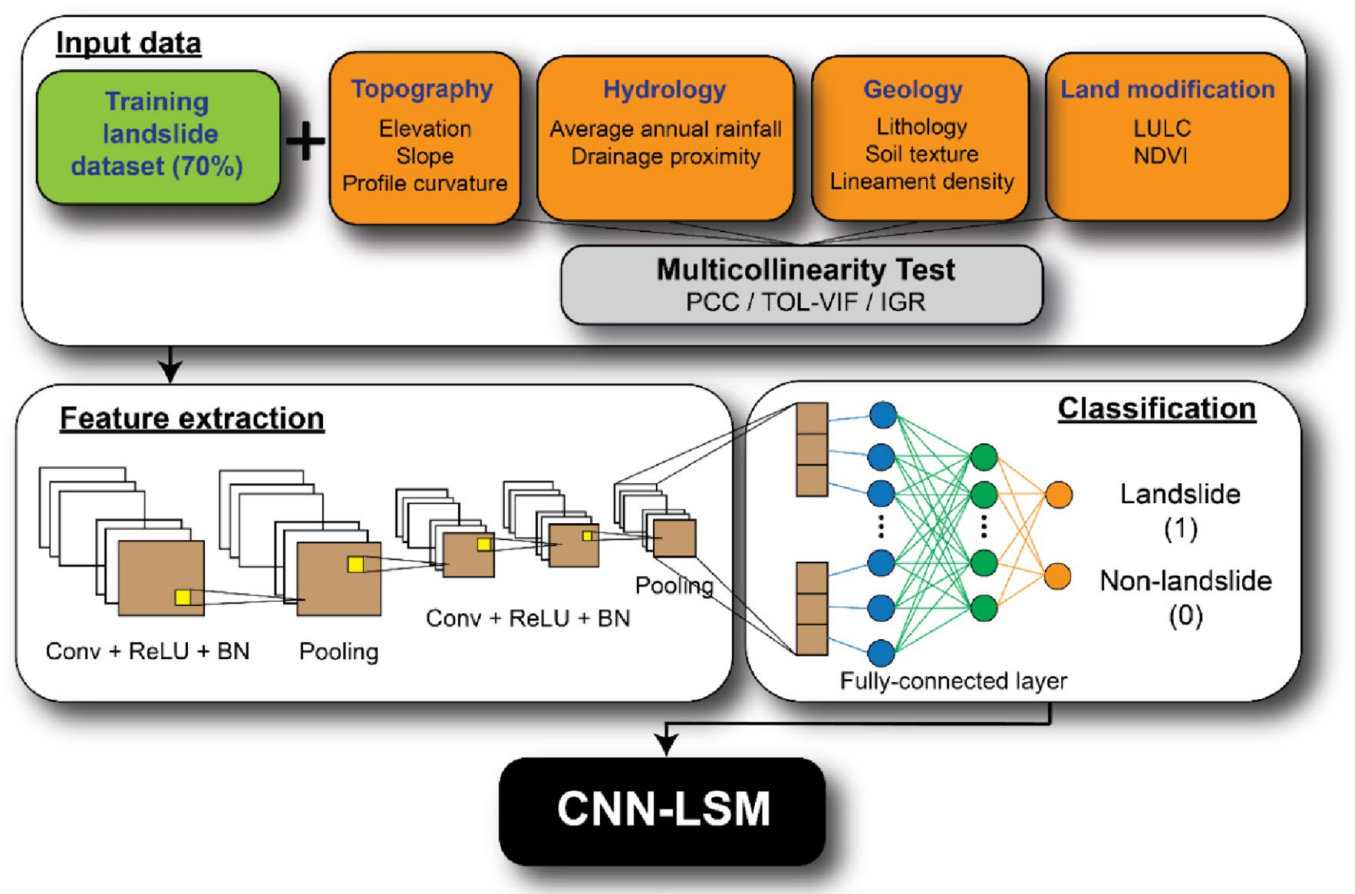
Workflow of the convolutional neural network (CNN)-based landslide susceptibility model, illustrating data preprocessing, feature extraction, model training, and prediction. Modified and redrawn from^115^, licensed under the Creative Commons Attribution 4.0 International License (CC BY 4.0; http://creativecommons.org/licenses/by/4.0/).

CNN architecture commonly consists of convolutional, pooling, and fully connected layers^113,114^. Convolutional layers apply learnable filters to identify meaningful feature combinations associated with landslide occurrence, followed by nonlinear activation functions such as Rectified Linear Unit (ReLU). Pooling layers reduce dimensionality and computational cost while improving robustness to minor spatial variability. The extracted features are then flattened and passed to fully connected layers, which integrate spatial information to predict landslide probability for each pixel. The final output size of the network is determined by the number of classes (landslide vs. non-landslide), and the number of layers is user-determined^111^. Adding more layers creates a deeper network capable of recognizing more complex features, producing a landslide susceptibility map^115^(Fig. 9).

Long Short-Term Memory (LSTM), a type of recurrent neural network (RNN), is increasingly used in DL for landslide susceptibility mapping, particularly for modelling the temporal aspects of landslide triggers. LSTM differs from traditional static models as they are specifically designed to capture long-term dependencies and complex nonlinear relationships within sequential data. This capability is crucial for LSM because the occurrence of a landslide is often influenced by the cumulative and delayed effects of various conditioning factors over time, not just their instantaneous values^115^.

**Fig. 10:**
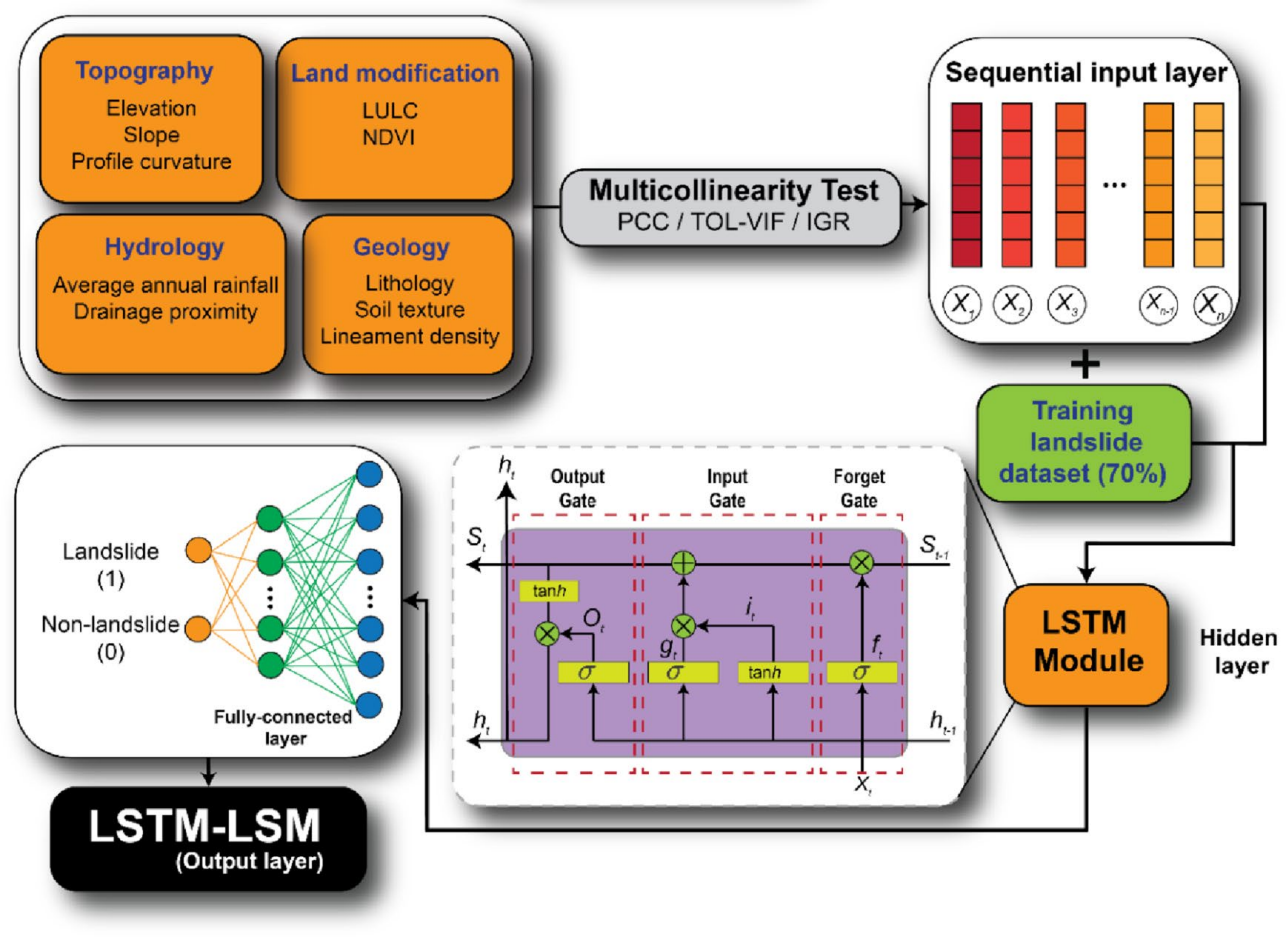
Workflow of the long short-term memory (LSTM)-based landslide susceptibility model, showing the input structure, sequence learning process, model training, and susceptibility prediction. Modified and redrawn from^115^, licensed under the Creative Commons Attribution 4.0 International License (CC BY 4.0; http://creativecommons.org/licenses/by/4.0/).

The core of an LSTM model’s power lies in its gating mechanisms, the input, forget, and output gates, which control the flow of information. The forget gate determines which past information from the “cell state” should be discarded, while the input gate decides which new information to store^115–117^. Finally, the output gate controls which data from the cell state is used to produce the final prediction (Fig. 10). This architecture allows the model to remember important information over long periods, effectively addressing the vanishing gradient problem that affects conventional RNNs.

#### Model parameter settings for ML and DL models

To ensure a consistent and reproducible comparison among models, parameter settings were standardized and optimized through preliminary testing and reference to previous landslide susceptibility studies. For the ML models, the RF architecture included 500 trees to ensure model stability, while the maximum tree depth was limited to prevent overfitting. The minimum number of samples required to split a node and the minimum number of samples per leaf were also controlled to improve generalization performance^106^. For the XGBoost model, key parameters included the learning rate (0.1), number of estimators (300), maximum tree depth (6), subsample ratio (0.8), and column sampling ratio (0.8). These parameters were selected to balance model complexity and computational efficiency while minimizing overfitting^109,110^.

For the DL models, the CNN architecture consisted of multiple convolutional layers, pooling layers, and fully connected layers with ReLU activation functions^113,114^. The LSTM model included hidden memory units that captured complex nonlinear relationships among conditioning factors. Both CNN and LSTM models were trained using the Adam optimizer with a learning rate of 0.001, batch size of 32, and 50 training epochs. Dropout regularization was applied to reduce overfitting^115^. All ML and DL models were trained on the same training dataset and evaluated on the same validation dataset to ensure a fair comparison within a unified, data-consistent framework.

### Model validation and accuracy assessment

Model validation was conducted using the area under the receiver operating characteristic (ROC) curve (AUC), ROC–AUC analysis, and confusion matrix metrics. By applying all models under identical input conditions, this study enables a controlled comparison, allowing differences in performance to be attributed to model behavior rather than inconsistencies in data or validation strategies. In addition, selected landslide locations were cross-validated using high-resolution satellite imagery and available field observations to ensure spatial reliability.

The ROC curve is a graphical plot that illustrates the diagnostic ability of a binary classifier system as its discrimination threshold varies. It plots the True Positive Rate (TPR) against the False Positive Rate (FPR). For landslide susceptibility, the TPR (also known as sensitivity or recall) represents the proportion of correctly predicted landslide pixels. In contrast, the FPR (1-specificity) is the proportion of non-landslide pixels incorrectly classified as landslide-prone pixels. The AUC value provides a single, aggregate measure of performance across all possible classification thresholds, with an AUC range of 0 to 1. An AUC value of 1.0 represents a perfect model, while a value of 0.5 indicates a model with no better predictive power than random chance. Moreover, an AUC value greater than 0.7 suggests good performance, and values above 0.8 are considered excellent. It is a widely accepted validation method as it is insensitive to data imbalance, a common issue in landslide datasets where non-landslide locations vastly outnumber landslide occurrences^118^.

**Fig. 11:**
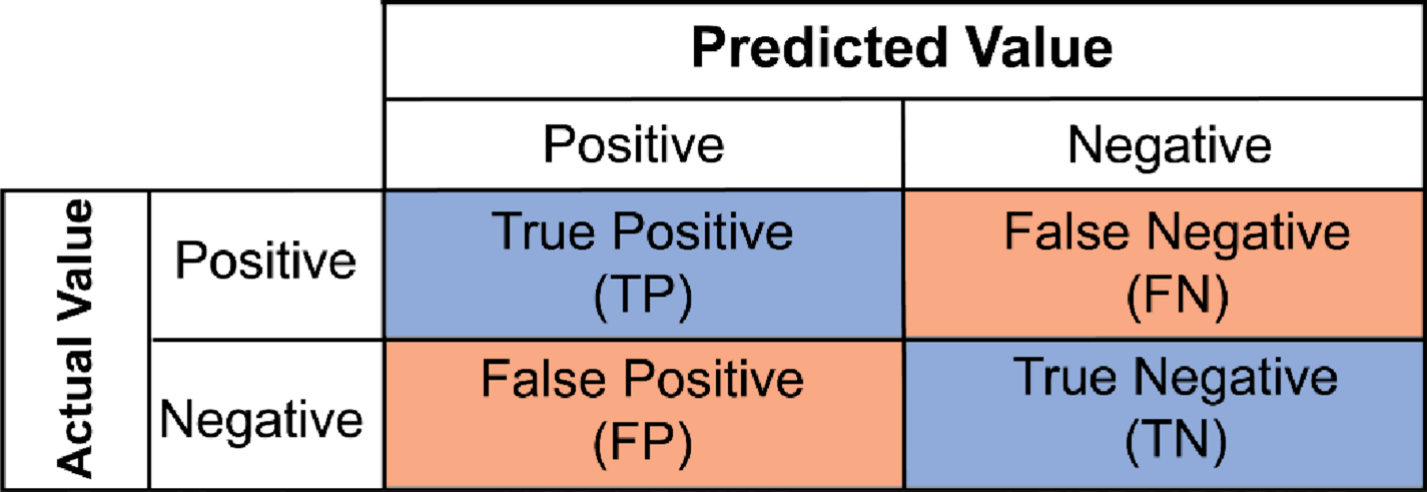
Confusion matrix used to evaluate the performance of landslide susceptibility models.

Validation of landslide susceptibility models can also be effectively conducted using confusion matrix metrics, such as the precision-recall area under the curve (PR AUC)^119^. While the ROC curve is a valuable tool, the PR curve is particularly informative for imbalanced datasets, which are common in landslide studies where the number of non-landslide points far exceeds that of landslide points. The PR curve plots precision against recall at various threshold settings^120^. Precision measures the proportion of correctly predicted positive cases (landslides) among all predicted positive cases. Recall, on the other hand, measures the proportion of actual positive cases that were correctly identified^121^. The F1-score is the harmonic mean of precision and recall, indicating that the model effectively identifies landslides while minimizing false positives and negatives. Accuracy measures the proportion of correctly classified landslide and non-landslide points, indicating the model’s overall performance^122,123^.

11 $$\:\mathrm{P}\mathrm{r}\mathrm{e}\mathrm{c}\mathrm{i}\mathrm{s}\mathrm{i}\mathrm{o}\mathrm{n}=\:\frac{\mathrm{T}\mathrm{P}}{\mathrm{T}\mathrm{P}+\mathrm{F}\mathrm{P}}$$

12 $$\:\mathrm{R}\mathrm{e}\mathrm{c}\mathrm{a}\mathrm{l}\mathrm{l}=\:\frac{\mathrm{T}\mathrm{P}}{\mathrm{T}\mathrm{P}+\mathrm{F}\mathrm{N}}$$

13 $$\:\mathrm{F}1-\mathrm{s}\mathrm{c}\mathrm{o}\mathrm{r}\mathrm{e}=2\:\mathrm{x}\:\frac{\mathrm{P}\mathrm{r}\mathrm{e}\mathrm{c}\mathrm{i}\mathrm{s}\mathrm{i}\mathrm{o}\mathrm{n}\:\mathrm{x}\:\mathrm{R}\mathrm{e}\mathrm{c}\mathrm{a}\mathrm{l}\mathrm{l}}{\mathrm{P}\mathrm{r}\mathrm{e}\mathrm{c}\mathrm{i}\mathrm{s}\mathrm{i}\mathrm{o}\mathrm{n}+\mathrm{R}\mathrm{e}\mathrm{c}\mathrm{a}\mathrm{l}\mathrm{l}}$$

14 $$\:\mathrm{A}\mathrm{c}\mathrm{c}\mathrm{u}\mathrm{r}\mathrm{a}\mathrm{c}\mathrm{y}=\frac{\mathrm{T}\mathrm{P}+\mathrm{T}\mathrm{N}}{\mathrm{T}\mathrm{P}+\mathrm{F}\mathrm{P}+\mathrm{F}\mathrm{N}+\mathrm{T}\mathrm{N}}$$

where $\:\mathrm{T}\mathrm{P}$ is the number of samples correctly predicted as positive, $\:\mathrm{F}\mathrm{P}$ refers to samples incorrectly predicted as positive (actual negative), $\:\mathrm{F}\mathrm{N}$ represents samples incorrectly predicted as negative (actual positive), and $\:\mathrm{T}\mathrm{N}$ indicates samples correctly predicted as negative^124,125^ (Fig. 11). The PR AUC provides a single value that summarizes the trade-off between precision and recall across all thresholds. A higher PR AUC indicates a better-performing model, especially in correctly identifying the rare positive class (landslides) without excessive false alarms. Given the nature of landslide data, the PR AUC is often considered a more reliable metric than ROC AUC, as it focuses specifically on the model’s performance on the minority class, offering a clearer picture of its practical utility^126^.

## Results

### Landslide inventory data

The landslide inventory consists of 1,752 sampling points, including 876 landslide and 876 non-landslide locations. Landslide data were compiled from the Department of Mineral Resources (DMR) and supplemented with aerial photographs, satellite imagery, and field surveys. Of these, 752 landslides were recorded as points and 124 as polygons, which were standardized to a polygon-based format with point centroids for modelling consistency. The mapped landslides range from approximately 500 to 20,000 m² and are predominantly shallow translational slides and debris flows, with depths of 0.5–3.0 m. These failures mainly occur in weathered materials composed of rock fragments and colluvial deposits on steep slopes, which can rapidly mobilize under intense rainfall.

The dataset was split into 70% for training (1,226 samples) and 30% for validation (526 samples). This structured dataset provides a representative basis for analyzing landslide susceptibility in the study area.

### Landslide conditioning factors (LCFs)

All 10 conditioning factors for landslides were examined and categorized into four groups: topographical, hydrological, geological, and land-modification-related factors (Table 2). For topographical factors, the analysis indicated that the elevation range of 433–948 m was prevalent (covering up to 46% of the range) and was associated with the highest landslide occurrence. In comparison, higher elevations (> 949 m) accounted for 25% of the total area but represented lower landslide occurrence. Steep slope gradients exceeding 30° accounted for only 8.2% of the terrain and 14% of the landslides, compared with moderately sloped gradients between 17–32°, which were responsible for almost 60% of the landslide occurrences. However, the ratio of landslide occurrence to slope classes showed that steep slopes were more likely to experience material movement. Additionally, the range was predominantly concave (covering 40% of the landscape) and prone to landslides (accounting for 47% of occurrences), while convex slopes accounted for 35% (Table 2).

In terms of hydrological factors, most annual rainfall ranged from 1,505 to 1,652 mm; higher elevations experienced higher rainfall. However, the rainfall exceeding 1,603 mm annually accounted for 44% of landslide occurrences, suggesting that higher rainfall was associated with a higher landslide risk. Furthermore, 80% of landslides occurred within 200 m of the drainage system, while only 20% took place at distances greater than 200 m (Table 2). This finding confirmed that the river can erode the base of slopes, leading to instability and landslides. Geologically, coarse-grained clastic sedimentary rock was the dominant lithology, accounting for 77% of the area. This rock type represented 75% of landslide occurrences. However, the ratio between rock type and landslide occurrence was highest in terrain covered by finer-grained sedimentary rock. The undifferentiated soil texture also played a significant role in landslide occurrences. It covered 90% of the landscape and was responsible for 98% of landslide events. The presence of erodible rocks and soil on steep slopes increased slope instability. On the other hand, the terrain with lower lineament density showed a higher landslide occurrence. 83.5% of landslides were observed in areas with low lineament density (Table 2). The highest frequency ratio was also found in the class of lower lineament density.

Regarding factors related to land modification, trees and forests predominated in the landscape, accounting for 61% of the total area and representing 55% of landslide incidents. The other land types modified for crops and plants accounted for approximately 45% of landslide occurrences but had the highest frequency ratio among LULC classes. Furthermore, a significant portion (91%) of landslide events was recorded in areas with moderate to high NDVI values, while 9% occurred in regions with limited vegetation cover (Table 2). This finding indicated that although the vegetation was mild, it might not adequately secure the vulnerable slopes. High vegetation density at higher elevations and on steep terrain tended to reduce the areas susceptible to landslides.

### Landslide susceptibility maps (LSMs)

#### Multicollinearity analysis

In this study, multicollinearity among the conditioning variables was assessed using Pearson’s correlation coefficient (PCC) (Table 3), tolerances (TOL), and variance inflation factors (VIF) (Table 4). The findings indicated that the highest correlation was 0.435 between soil texture and lithology. However, this value was below 0.7, which signifies that high collinearity was not present. For TOL and VIF, the results showed that the lowest TOL was 0.632 and the highest VIF was 1.582. These values were within acceptable critical thresholds (TOL < 0.10 and VIF < 10), indicating no multicollinearity among the 10 conditioning factors.

**Fig. 12:**
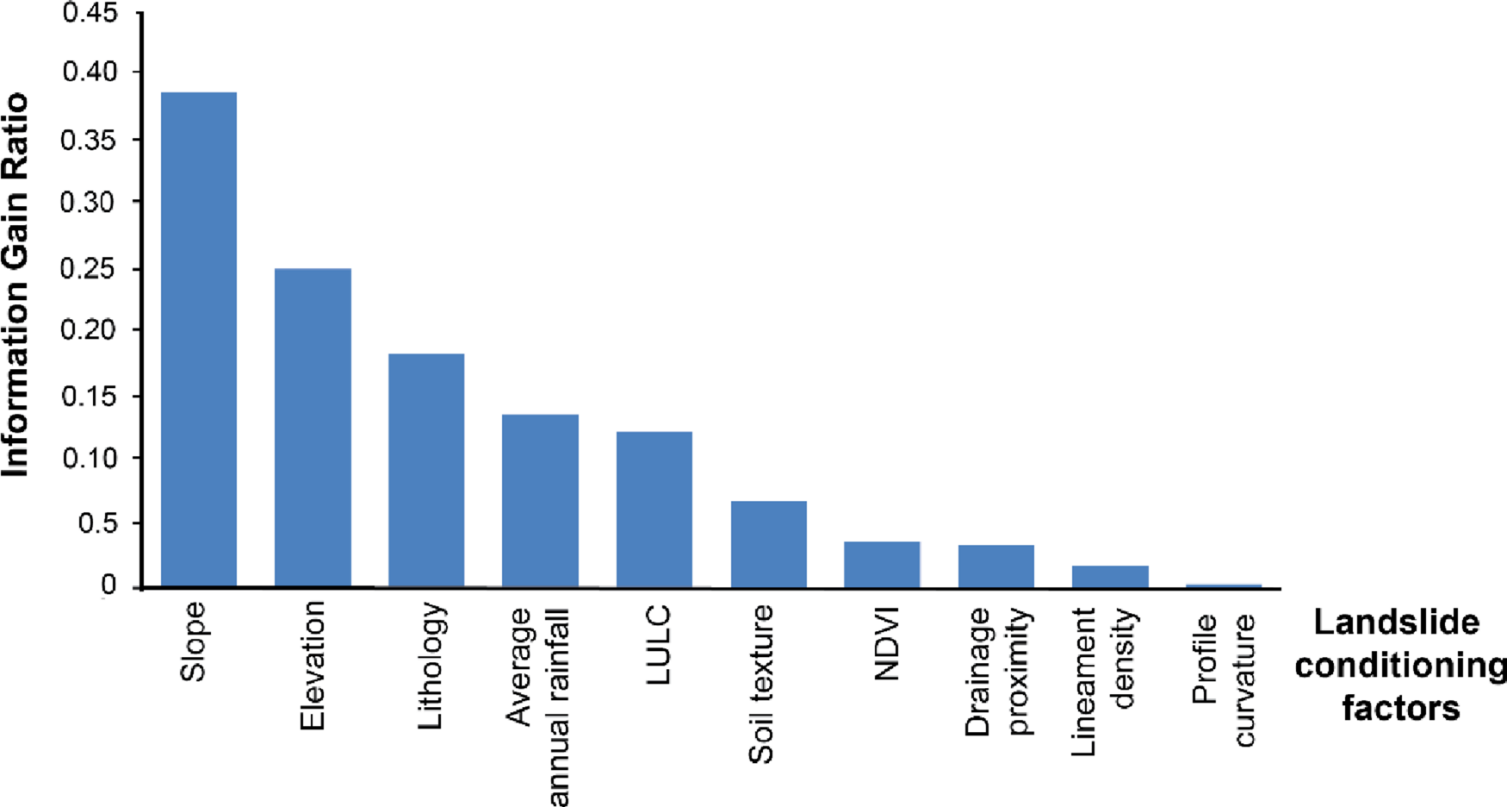
Relative importance of landslide conditioning factors determined using the Information Gain Ratio (IGR) method.

The results of the variable importance analysis in the feature analysis procedure were presented in Table 4. Although all conditioning factors contributed to the model performance, slope emerged as the most influential factor (0.387), followed by elevation (0.250), lithology (0.183), average annual rainfall (0.136), and LULC (0.123) (Fig. 12). The dominant role of slope was attributed to the concentration of landslide occurrences at sloped terrain greater than 17^o^, which accounted for approximately 73% of the recorded landslide events. Similarly, the importance of the elevation range between 430 and 950 m reflected the spatial cluster of landslides, which accounted for about 66% of the total area. The remaining conditioning factors were presented in terms of their relative importance: soil texture (0.069), NDVI (0.037), drainage proximity (0.034), lineament density (0.018), and profile curvature (0.004), respectively (Fig. 12). Combining all multicollinearity tests, however, all 10 conditioning factors were determined to be independent and suitable for landslide susceptibility mapping.

**Table 2:** Landslide conditioning factors and the frequency ratio values.

Categories	Factors	Classes	LCFs information				Frequency ratio (b/a)
			Class pixel	% Class pixel^a^	Landslide pixel	% Landslide pixel^b^	
Topography	Elevation (m)	143–432	9,042,289	27.64	129	10.97	0.40
		**433–690**	**7**,**664**,**952**	**23.43**	**401**	**34.10**	**1.46**
		691–948	7,674,812	23.46	374	31.80	1.36
		949-1,242	5,570,337	17.03	171	14.54	0.85
		1,243-1,947	2,757,196	8.43	101	8.59	1.02
	Slope (^o^)	0–8.9.9	5,879,039	17.99	83	7.06	0.39
		9.0–16.9.0.9	8,181,125	25.04	229	19.47	0.78
		17.0–24.2.0.2	8,918,553	27.29	347	29.51	1.08
		24.3–32.8	7,011,194	21.46	354	30.10	1.40
		**32.9–81.3**	**2**,**685**,**380**	**8.22**	**163**	**13.86**	**1.69**
	Profile curvature	**Concave**	**12**,**960**,**740**	**39.62**	**556**	**47.28**	**1.19**
		Flat	6,809,289	20.82	212	18.03	0.87
		Convex	12,939,557	39.56	408	34.69	0.88
Hydrology	Average annual rainfall (mm/yr)	1,410-1,504	3,029,916	9.26	81	6.89	0.74
		1,505-1,554	6,604,330	20.19	147	12.50	0.62
		1,555-1,602	9,852,124	30.12	423	35.97	1.19
		**1**,**603-1**,**652**	**7**,**405**,**627**	**22.64**	**362**	**30.78**	**1.36**
		1,653-1,763	5,817,728	17.79	163	13.86	0.78
	Drainage proximity (m)	**0–200**	**25**,**897**,**615**	**79.17**	**947**	**80.53**	**1.02**
		201–400	5,910,869	18.07	203	17.26	0.96
		401–600	814,277	2.49	24	2.04	0.82
		601–800	84,895	0.26	2	0.17	0.66
		810-1,185	2,087	0.01	0	0.00	0.00
Geology	Lithology	Quaternary sediment	2,968,027	9.12	11	0.94	0.10
		Bedded limestone	139,861	0.43	1	0.09	0.20
		**Fine-grained sedimentary rock**	**3**,**247**,**970**	**9.98**	**220**	**18.79**	**1.88**
		Coarse-grained sedimentary rock	25,222,575	77.46	881	75.23	0.97
		Granitic rock	981,644	3.01	58	4.95	1.64
	Soil texture	Well-drained sand	470,455	1.45	3	0.26	0.18
		Moderately-drained sand	2,370,497	7.32	11	0.94	0.13
		Poorly-drained sand	120,698	0.37	1	0.09	0.23
		Poorly-drained silt and clay	574,354	1.77	1	0.09	0.05
		**Soil on steep slopes**	**28**,**862**,**261**	**89.09**	**1151**	**98.63**	**1.11**
	Lineament density (km/km^2^)	0–0.14.14	27,039,897	82.67	982	83.50	1.01
		**0.15–0.42**	**2**,**197**,**057**	**6.72**	**95**	**8.08**	**1.20**
		0.43–0.70	2,662,091	8.14	87	7.40	0.91
		0.71–1.04	505,706	1.55	6	0.51	0.33
		1.05–1.79	304,974	0.93	6	0.51	0.55
Land modifications	LULC	Water body	96,478	0.30	1	0.09	0.29
		Forest	19,962,127	61.61	642	55.01	0.89
		Urban area	215,954	0.67	1	0.09	0.13
		Shrubland	297,089	0.92	1	0.09	0.09
		**Agricultural land**	**11**,**826**,**618**	**36.50**	**522**	**44.73**	**1.23**
	NDVI	−0.30-0	51,178	0.16	1	0.09	0.54
		0–0.20.20	1,893,111	5.79	80	6.81	1.18
		**0.21–0.40**	**7**,**300**,**246**	**22.32**	**356**	**30.30**	**1.36**
		0.41–0.60	22,864,761	69.90	726	61.79	0.88
		0.61–0.82	600,330	1.84	12	1.02	0.56

**Bold numbers** are the highest frequency ratio (FR) of each landslide conditioning factor.

**Table 3:**
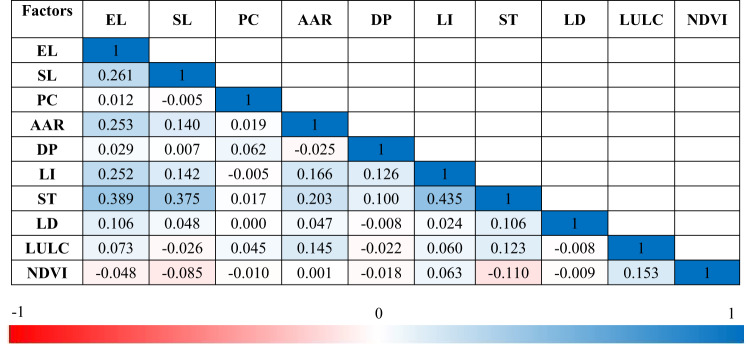
Pearson’s correlation coefficient (PCC).

Note EL: elevation, SL: slope, PC: profile curvature, AAR: average annual rainfall, DP: drainage proximity, LI: lithology, ST: soil texture, LD: lineament density, LULC: land use and land cover, NDVI: normalized difference vegetation index.

**Table 4:** Tolerances (TOL), variance inflation factors (VIF), and information gain ratio (IGR).

Categories	Landslide conditioning factors	Collinearity statistics		Feature importance
		TOL	VIF	IGR
Topography	Elevation	0.794	1.260	0.250
	Slope	0.832	1.202	0.387
	Profile curvature	0.993	1.007	0.004
Hydrology	Average annual rainfall	0.900	1.112	0.136
	Drainage proximity	0.972	1.028	0.034
Geology	Lithology	0.776	1.288	0.183
	Soil texture	0.632	1.582	0.069
	Lineament density	0.982	1.019	0.018
Land modifications	LULC	0.932	1.073	0.123
	NDVI	0.943	1.061	0.037

#### Landslide susceptibility analysis

**Fig. 13:**
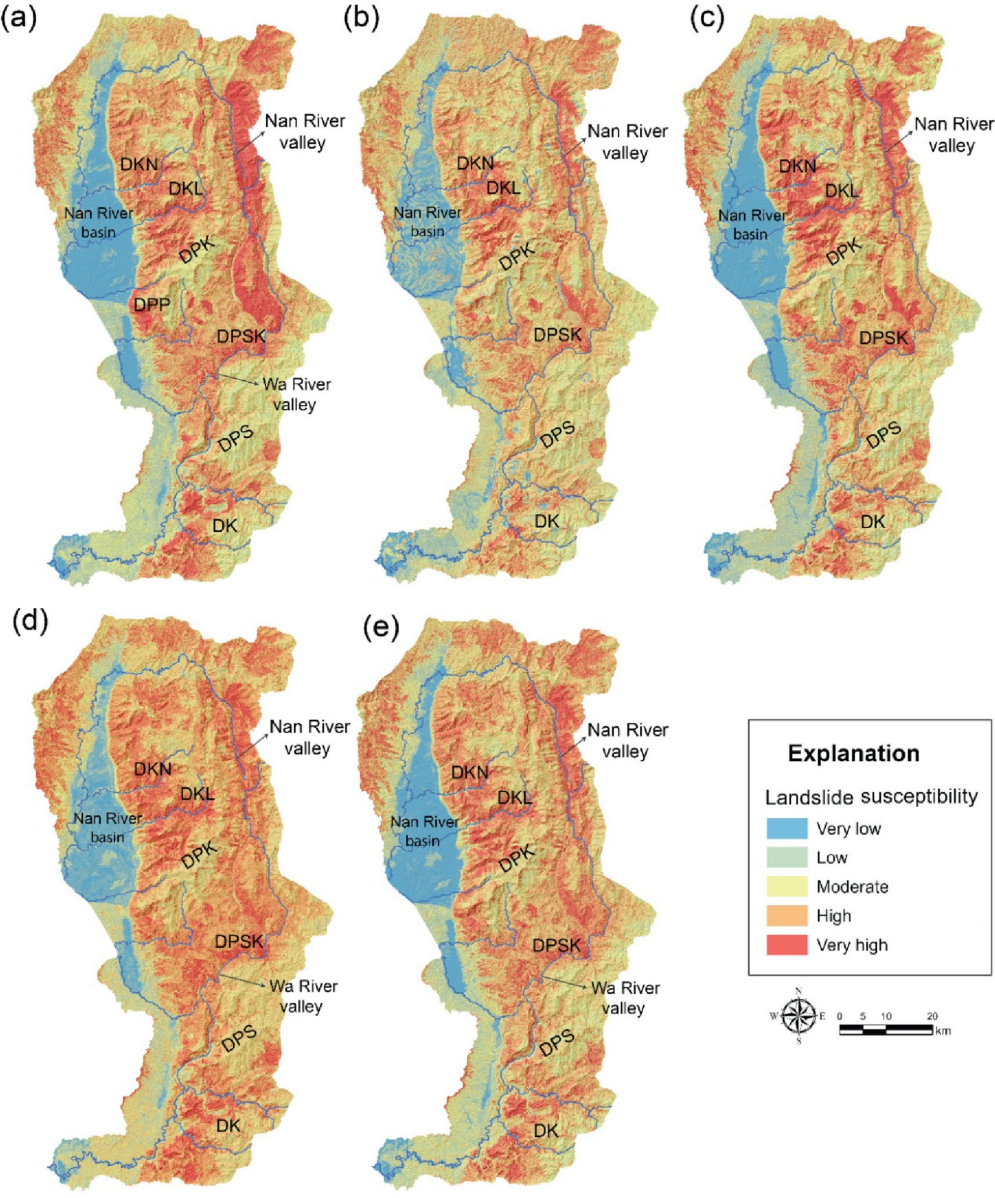
Landslide susceptibility maps produced using **(a)** Frequency Ratio (FR), **(b)** Random Forest (RF), **(c)** XGBoost, **(d)** CNN, and **(e)** LSTM models.

Combining all 10 LCFs with the training dataset, the LSMs derived from FR, ML-based (RF and XGBoost), and DL-based (CNN and LSTM) approaches were shown in Fig. 10. For the FR model, the factor ratio values for all 10 factors were summarized in Table 2. FR values close to 0 indicated low landslide potential, whereas FR values greater than 1 reflected higher susceptibility. The higher FR values suggested that these sub-classes exerted stronger control over landslide occurrence. The FR-based susceptibility map was divided into five classes using the natural break classification scheme (Fig. 13a). The high and very high susceptibility categories accounted for 51% of the area along the Nan River and Wa River valleys in the east, and most of DKN, DKL, DPSK, and west of DK. 28.44% of the study area fell within the moderate classes, distributed throughout the range’s hills. Approximately 10% were categorized in both low- and very-low susceptibility classes observed in the western Nan River basin (Table 5).

As the LSI was classified using the natural break method, the ML-based approaches, including RF and XGBoost models, indicated 11% and 16% in the very high susceptible class, respectively. These very high classes were distributed in the Nan River valley in the east, DNK, and DKL. The highly susceptible class was along the foothills of the range, accounting for > 30% of the study site. The moderately susceptible classes were distributed across most hilltops in Doi Phu Kha (DPK), DPSK, DSPS, and DK. The combined low-to-very-low susceptible classes were observed only in the western Nan River basin and small intermontane valleys, which together accounted for approximately 20% of the total study site from both models (Fig. 13b-c; Table 5).

For the DL-based approaches, the study classified LSI using a natural-break classification scheme and identified the most susceptible classes as those with the highest proportions, achieving 43% and 36% coverage with CNN and LSTM, respectively. The very high class was approximately 18% in both models, observed along the Nan River valley in the east, the Wa River valley, across the high mountains of DKN, DKL, and DPSK, and west of DK, followed by the moderate class at around 25% along hillslopes. The Nan River basin in the west revealed the lower-susceptible classes, accounting for 15% of the total study area (Fig. 13d-e; Table 5).

**Table 5:** Landslide susceptibility classification with area coverage and percentages for five predictive models.

Types/ Models Classes	Bivariate statistic		Machine learning				Deep learning			
	FR		RF		XGBoost		CNN		LSTM	
	Area (km^2^)	%	Area (km^2^)	%	Area (km^2^)	%	Area (km^2^)	%	Area (km^2^)	%
Very low	457.55	9.08	354.10	7.02	511.50	10.15	288.65	5.73	456.66	9.06
Low	545.43	10.82	599.57	11.89	627.10	12.44	469.91	9.32	450.77	8.94
Moderate	1,433.80	28.44	1,819.64	36.10	1,452.14	28.81	1,196.22	23.73	1,445.55	28.68
High	1,717.62	34.07	1,682.46	33.38	1,636.58	32.47	2,146.60	42.58	1,817.30	36.05
Very high	886.65	17.59	585.26	11.61	813.73	16.14	939.66	18.64	870.76	17.27

**Fig. 14:**
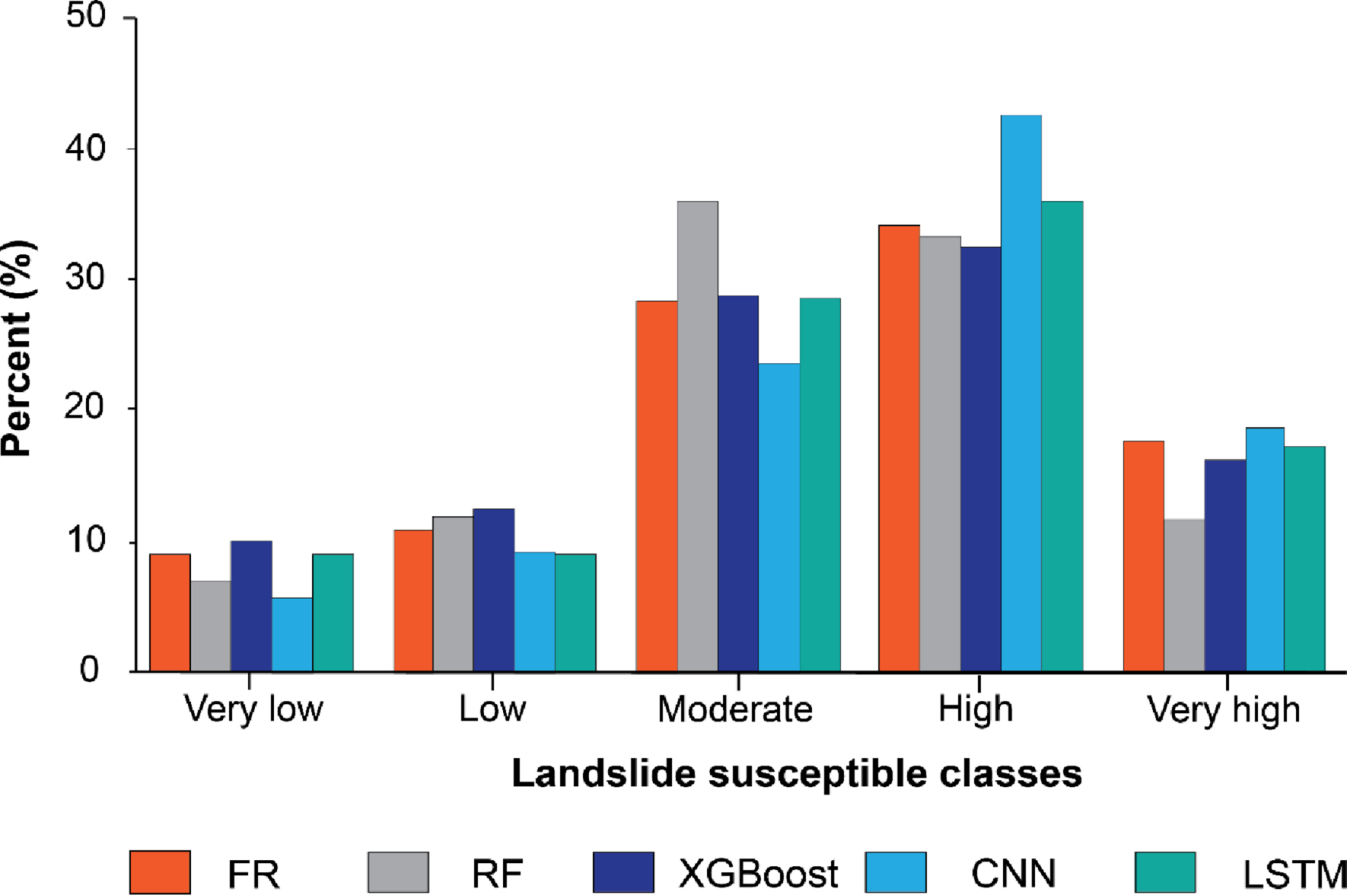
Distribution and percentage of landslide susceptibility classes derived from the five models.

The landslide susceptibility classes derived from each model were compared (Table 5). They showed that most of the study areas were classified as moderate to high, with CNN showing the highest percentage in the high susceptibility class, at over 40%. In contrast, all models consistently assigned the lowest percentages to the low-to-very-low categories, typically below 15%. Although there was general agreement on the distribution of susceptibility, there were slight variations. For instance, the RF model identified the largest proportion of moderately susceptible areas. At the same time, CNN and LSTM identified higher landslide-susceptible classes than the traditional FR model. However, the XGBoost model classified all landslide-susceptible categories similarly to the FR model (Fig. 14).

#### Model validations

**Fig. 15:**
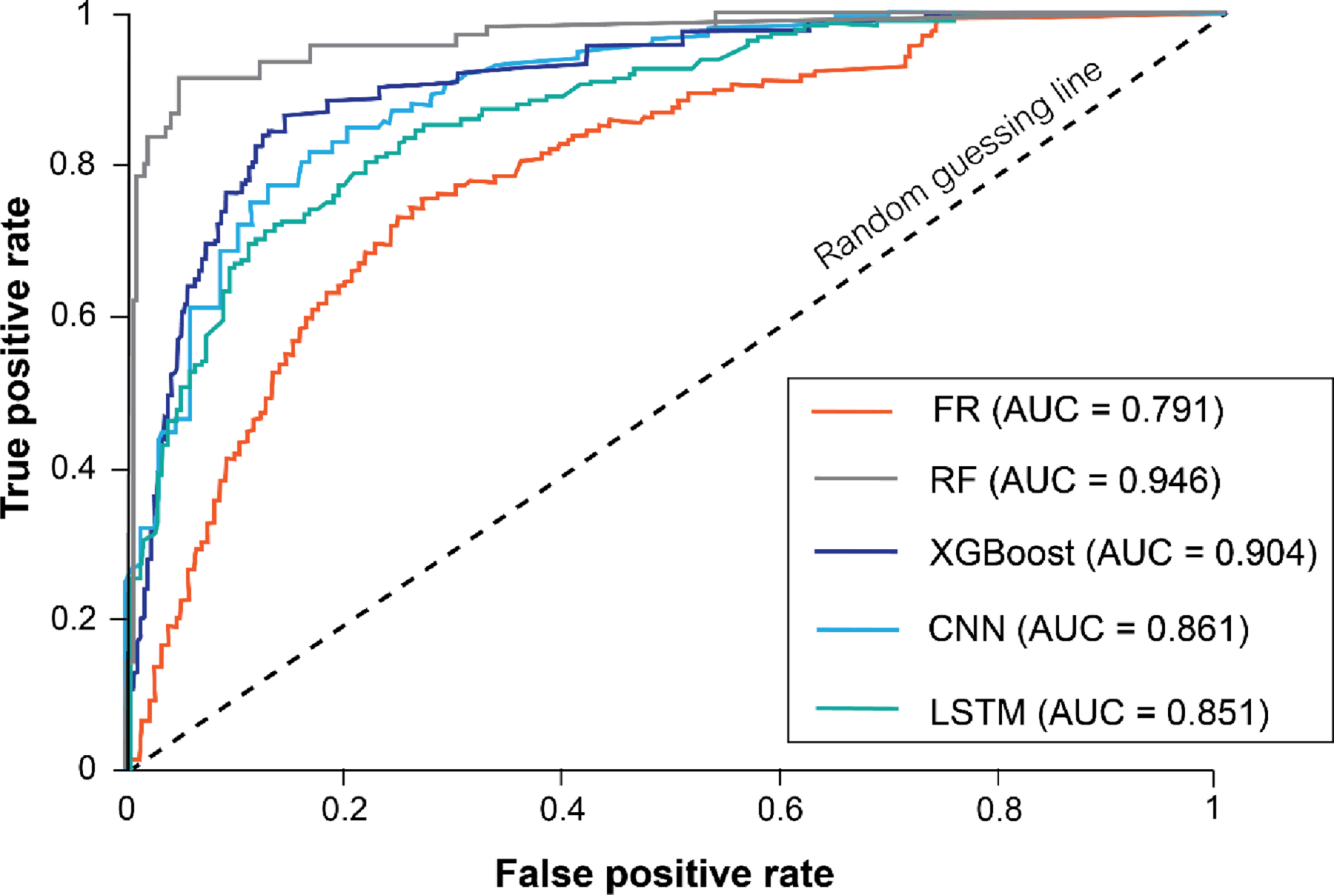
Receiver operating characteristic (ROC) curves and area under the curve (AUC) values for model performance evaluation.

Model performance was evaluated using ROC AUC on the validation dataset^127,128^ (Table 6). The ROC curves for all models were close to the upper-left corner, indicating strong predictive performance (Fig. 15). The RF model had the highest AUC of 0.946, indicating that 94.6% of landslide pixels are correctly classified as landslide. XGBoost, CNN, and LSTM achieved AUC values of 0.904, 0.861, and 0.851, respectively (Fig. 15). The bivariate statistic model for FR achieved an AUC of 0.791. Compared to all models, the RF model achieved the highest accuracy, while the FR model achieved the lowest. However, the AUC classification results showed that all models demonstrated high accuracy and reliability in identifying landslide susceptibility across the range^129^.

The ML- and DL-based LSMs obtained from RF, XGBoost, CNN, and LSTM were further evaluated using the area under the precision-recall curve (PR AUC), including precision, recall, F1-score, and overall accuracy (Table 6). Given optimal thresholds between 0.5 and 0.7, all models achieved precision above 0.8, with XGBoost achieving the highest (0.876), followed by CNN (0.870) and RF (0.838). The high precision indicated that more than 80% of predicted landslide occurrences were correctly classified. Recall values were also robust, exceeding 0.90 for both RF and XGBoost. The values suggested that over 90% of actual landslide events were successfully detected. The recall values were lower in CNN and LSTM-based models. The F1-scores of 0.812 (RF), 0.876 (XGBoost), 0.785 (CNN), and 0.702 (LSTM) indicated effective model performance, with a balance between precision and recall. In addition, RF, XGBoost, CNN, and LSTM models achieved overall accuracies of 0.889, 0.927, 0.802, and 0.734, respectively, demonstrating reliable landslide-susceptibility prediction across the range (Table 6).

**Table 6:** Validation metrics, including precision, recall, F1-score, and accuracy for the RF, XGBoost, CNN and LSTM.

Model	Precision	Recall	F1-score	Accuracy
RF	0.838	0.947	0.812	0.889
XGBoost	0.876	0.984	0.876	0.927
CNN	0.870	0.743	0.785	0.802
LSTM	0.821	0.664	0.702	0.734

## Discussion

### Correlation between LCFs

The LSMs were developed from the bivariate statistics, ML (including RF, and XGBoost), and DL-based (CNN and LSTM) approaches utilized all 10 LCFs, which are classified into four categories: topography (elevation, terrain slope, and profile curvature), hydrology (average annual rainfall and proximity to drainage), geology (lithology, soil texture, and lineament density), and land modification (land use/land cover, and NDVI).

The significance of each conditioning factor to the occurrence of landslides was determined using the information gain ratio (IGR). By selecting factors with high contributions, IGR can increase the precision of the model results^130^. Using IGR, the study identified slope, elevation, lithology, average annual rainfall, and LULC as the key factors for landslide occurrence along the range. The IGR is considered more reliable because it mitigates this bias and provides a comprehensive evaluation of the elements across multiple datasets^96^. Moreover, the conditioning factors with lower significance in influencing landslide occurrence include soil texture, NDVI, proximity to drainage systems, lineament density, and profile curvature (Fig. 12).

In this study, slope gradient is a primary controlling factor of landslide occurrence in the study area, where steep terrain, often exceeding 30°, acts on thick layers of weathered residual soil and colluvium, reducing slope stability and increasing the likelihood of failure^59^. In combination with this geomorphic setting, intense monsoonal rainfall (> 1,500 mm/year) plays a critical role by increasing soil moisture, elevating pore-water pressure, and weakening the soil–bedrock interface, ultimately triggering landslides^131,132^. The predominance of layered sedimentary bedrock overlain by a thick weathered mantle further enhances susceptibility^133^. The progressive saturation reduces shear strength and facilitates the development of failure planes^134,135^. Elevation also influences landslide distribution by controlling topographic complexity and climatic variables^14^. Moreover, land-use changes, such as deforestation and agricultural expansion, contribute to slope destabilization and increased exposure to erosion^136^.

In this study, long-term average annual rainfall is used to represent general hydroclimatic conditions and their interactions with vegetation cover and lithology. However, this approach does not explicitly capture short-term rainfall variability or event-based triggering mechanisms, which are critical drivers of landslides in tropical mountainous regions. Therefore, the results should be interpreted as representing relative susceptibility patterns rather than specific rainfall thresholds for failure initiation^137^. This limitation reflects the fundamental distinction between susceptibility mapping and early warning analysis. Incorporating event-based rainfall data and intensity–duration relationships may provide more robust triggering thresholds and improve the temporal accuracy of landslide prediction in monsoon-dominated environments.

Furthermore, while monsoonal rainfall patterns are considered, this study does not account for potential changes in future climate conditions. In Northern Thailand, climate change is expected to alter rainfall intensity, frequency, and seasonality, potentially increasing the occurrence of extreme rainfall events. Such changes may increase slope saturation, elevate pore-water pressure, and lead to more frequent or larger landslides. Consequently, the susceptibility patterns identified in this study represent present-day conditions rather than future hazard scenarios. Incorporating climate projections and extreme rainfall data would provide a more comprehensive understanding of long-term landslide risk.

Moreover, strong correlations are also observed between landslide occurrence and other less influential factors. The prominence of soil texture highlights the role of local geomorphic processes. Landslides predominantly occur within undifferentiated soil and/or mixed surficial deposits on steep slopes. The slope failures are primarily governed by poorly drained, cohesion-loss, and saturation-driven mechanisms during intense rainfall events^133^. In addition, factors such as drainage proximity and lineament density are notable influences on landslide distribution in the study area, in agreement with numerous studies^138,139^. While proximity to drainage networks increases landslide risk by promoting toe erosion, removing basal support, and enhancing soil saturation from adjacent water bodies, thereby decreasing shear strength, lineament density derived from clusters of fractures and faults represents zones of structural weakness that facilitate increased water infiltration, decrease rock strength, and induce mass movement on the slope. Notably, an environmental factor, such as NDVI, is a less influential conditioning factor in the range. Although vegetation influences soil moisture through processes such as transpiration, rainfall redistribution, and root development, NDVI may not adequately capture these complex interactions in the mountainous region, where NDVI variability is relatively limited to detect^70^. LULC better captures vegetation effects that contribute to landslide occurrence^140^. However, neither NDVI nor LULC emerges as a dominant factor compared to the other factors. Although these factors are less relevant to this study, they can also align with other local conditions to enhance landslide-susceptibility prediction in the mountain range.

It is important to note that the study relies primarily on environmental proxy variables (i.e., topography, land use, and rainfall) rather than direct geotechnical measurements such as soil cohesion or pore-water pressure^141^. While these physical parameters play a fundamental role in controlling slope stability, they are rarely available at regional scales due to the difficulty of field measurement, particularly in mountainous and remote areas. As a result, most LSMs adopt proxy-based approaches to represent the combined effects of geological, hydrological, and geomorphological processes.

Because proxy variables are used rather than physical reality, the results were interpreted as relative susceptibility patterns rather than the deterministic predictions of landslide occurrence. Despite this limitation, proxy-based models remain effective for regional-scale hazard assessment and land-use planning, where spatial coverage and consistency are critical. Future research should integrate geotechnical parameters, such as soil strength and pore-water pressure, with data-driven approaches to enhance the physical interpretability and predictive capability of landslide models.

### Model comparisons

The LSMs generated by all the models display consistent spatial patterns. Figure 13 shows that high- and very-high-susceptibility classes are primarily concentrated in the Nan River valleys in the east and in most mountainous areas of DKN, DKL, and DPSK, and in the west of DK. These susceptible classes are also distributed across higher-elevation areas in FR-, CNN-, and LSTM-based models. The moderately susceptible class is along hillslopes in the elevated terrain. The low-susceptible zones are consistently associated with low-elevation regions of the Nan River basin.

The comparative evaluation of the FR, RF, XGBoost, CNN, and LSTM models highlights apparent differences in their ability to create LSMs along the range, with higher precision and accuracy. The FR model is based on statistical relationships between landslide events and conditioning factors. The model sometimes oversimplifies results and is unable to directly address nonlinear relationships and complex interactions among various landslide conditioning factors, resulting in less accurate predictions in complex terrains and across broad regions.

ML enhances the FR method for landslide prediction by using FR’s statistical outputs as inputs and applying algorithms to produce more reliable, automated, and efficient susceptibility maps. ML methods can learn complex patterns, find nonlinear relationships among factors, and improve accuracy beyond traditional GIS-based approaches. ML models overcome FR’s manual limitations, handle large datasets more effectively, capture intricate factor interactions, and provide superior confusion matrix metrics^142,143^. This study has demonstrated significantly higher AUC values for the RF, XGBoost, and FR models (Fig. 15).

DL generally outperforms traditional ML in landslide mapping by automatically extracting complex features from large, unstructured remote sensing data, achieving higher accuracy in identifying landslide-prone areas and in detailed susceptibility mapping. However, the study shows lower accuracy and precision for DL than for ML methods in landslide susceptibility modeling (Table 6; Fig. 15). Possible reasons include the limited training dataset, which is restricted to historical landslide scars detected from aerial photography, satellite imagery, and traditional field investigations. This dataset is inadequate and cannot capture the spatial-temporal complexity of landslide scars^144^. In data-scarce conditions, DL tends to overfit LSMs^145^. The ML methods are more robust, data-efficient, and better-suited to structured geomorphological factors commonly used in GIS-based analyses. They often result in more stable and reliable predictions at basin or regional scales.

The results of this study are consistent with recent studies demonstrating the effectiveness of ML and DL approaches for capturing complex nonlinear relationships in large datasets. Previous studies have shown that advanced models, including hybrid and ensemble frameworks, can improve predictive accuracy and model robustness by integrating the complementary strengths of different algorithms^146–148^. These approaches enhance the representation of spatial patterns and interactions among conditioning factors, particularly in complex and heterogeneous mountainous environments^146,147^. In addition, ML models such as Random Forest and boosting algorithms have been widely reported to outperform traditional statistical methods because they can model nonlinear relationships more effectively^147^. However, the differences in the model’s performance are attributed not only to the algorithms but also to the parameter configuration and data representation^146–148^.

### Limitations of models and the potential of ensembles

Although the models show strong predictive performance, their results should be interpreted with caution because model behavior is influenced not only by the algorithms but also by parameter configuration. Previous studies have emphasized that the reliability of landslide susceptibility models is strongly influenced not only by the selection of modelling algorithms but also by the quality and reliability of landslide and non-landslide datasets^43^. Other studies have shown that in locally weighted learning (LWL), the selection of neighborhood size significantly affects the balance between capturing local variability and maintaining model stability^149^. Similarly, in multilayer perceptron (MLP) models, parameters such as the number of hidden layers, neuron density, and learning rate control the balance between underfitting and overfitting, directly influencing model performance. Ensemble techniques have been shown to improve model stability and reduce prediction errors by combining multiple learning strategies^150^. This study’s findings confirm that differences in model performance may not solely reflect algorithms but also be influenced by data structure, sampling design, the spatial distribution of non-landslide samples and parameter configuration, highlighting the importance of data consistency and reliability in landslide susceptibility modelling.

In addition to parameter sensitivity, the reliability of landslide susceptibility models is strongly affected by data quality, particularly the completeness of the landslide inventory and the selection of non-landslide samples. Uncertainties in sampling strategies can significantly influence model outcomes and may lead to overestimated predictive accuracy. In this study, data reliability was improved through cross-validation of multiple sources, including satellite imagery and available field verification. Furthermore, while RF, XGBoost, CNN, and LSTM demonstrate strong predictive capability, each model has inherent limitations in capturing the full complexity of nonlinear landslide processes. For example, CNN is effective for spatial feature extraction, whereas LSTM is better suited for temporal dynamics. Neither approach alone can fully represent coupled spatiotemporal interactions. Although RF showed high stability and data efficiency in this study, challenges related to model uncertainty, data dependency, and limited interpretability remain.

In this context, ensemble-based deep learning neural network (DLNN) approaches provide a promising direction for future research. By integrating the strengths of multiple models, these hybrid frameworks can also better capture complex nonlinear relationships, such as interactions among terrain, tectonics, and monsoonal rainfall, thereby improving prediction accuracy and reducing spatial uncertainty [151–153]. However, such approaches typically require large, high-quality datasets and high computational cost, which may limit their applicability in data-constrained regions such as Northern Thailand.

It is important to emphasize that the novelty of this study lies not in developing a new algorithm, but in establishing a unified and data-consistent evaluation framework that systematically compares statistical, machine learning, and deep learning models under identical conditions. This framework enables a more reliable assessment of model robustness, uncertainty, and interpretability. Future research should build upon this foundation by incorporating ensemble approaches while carefully balancing predictive performance, data availability, and model interpretability.

### Spatial distribution of soil loss and geomorphic controls

The spatial distribution of soil loss rate has a strong correlation with landslide occurrence^154^. The expression highlights a significant relationship between topographic complexity and erosional intensity in the range. Soil loss is modeled using the Revised Universal Soil Loss Equation (RUSLE), which accounts for rainfall erosivity (R), soil erodibility (K), slope steepness and length (LS), land cover management (C), and conservation practices (P)^155–156^. Applying RUSLE in the range, the study reveals that very low to low soil loss categories are predominantly localized within the expansive valley floors of the western plains of the Nan River basin and the Wa River valley. In contrast, a marked transition to high-to-very high surface erosion rates is observed across the rugged central and eastern sectors, particularly in the upland mountainous domains of DKN, DKL, and DPSK (Fig. 14). This pattern indicates that the convergence of steep gradients and high surface runoff potential in these elevated zones accelerates sediment yield and land degradation. Furthermore, high erosion rates in these upland watersheds are frequently associated with highland agricultural cultivation, including corn, cassava, rubber, and sugarcane^157^.

**Fig. 16:**
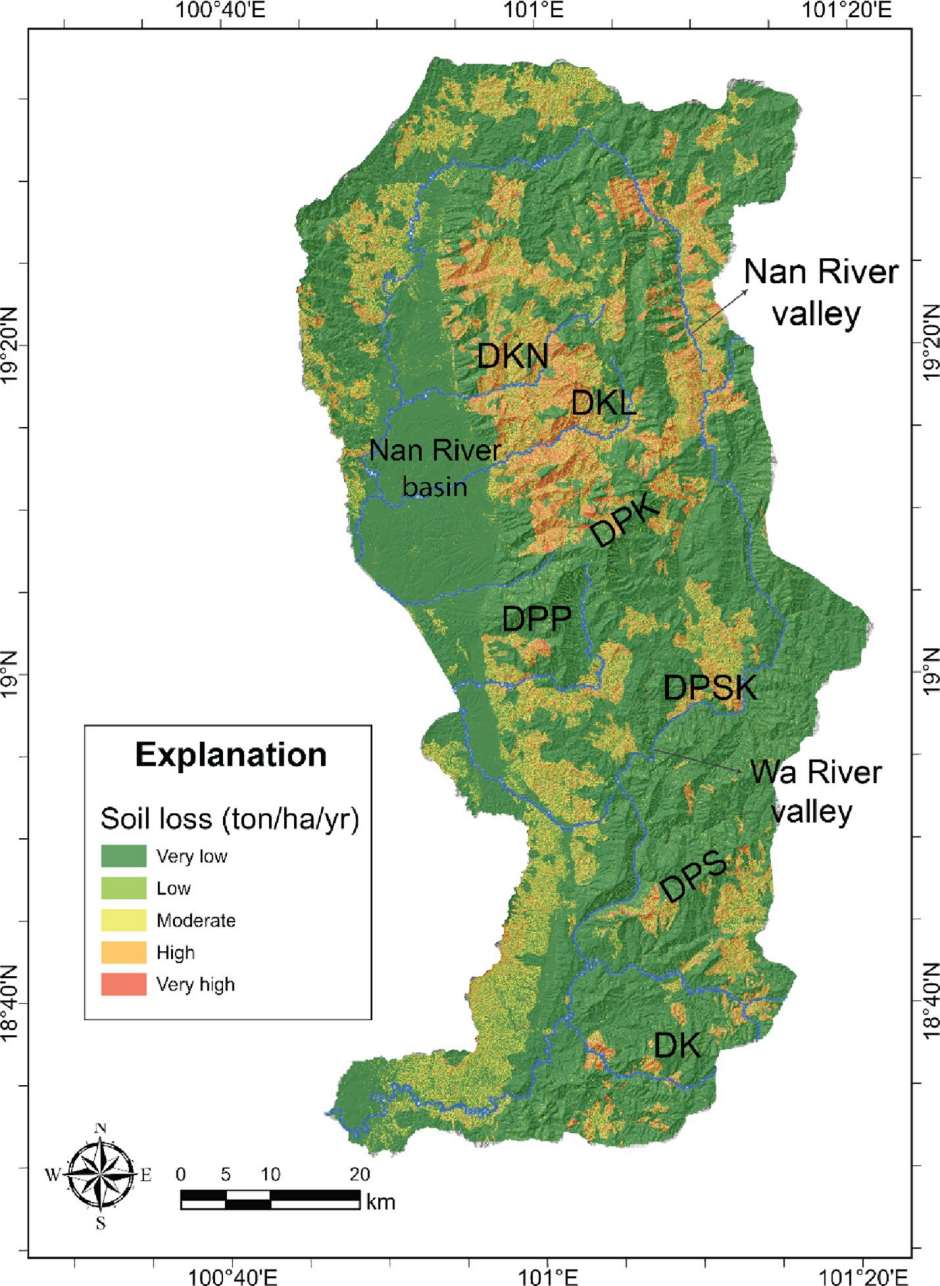
Spatial distribution of soil loss rates in the Luang Prabang Range (LPR).

Geomorphic heterogeneity further modulates the intensity of these erosional processes, as evidenced by the fragmented distribution of soil loss across distinct topographic features. While the DPP and DPSK sectors exhibit mixed signatures ranging from moderate to high, the DK in the southernmost portion of the study area presents a distinct cluster of high soil loss (Fig. 16). These variations suggest that localized drainage density and the structural orientation of specific mountain ranges exert a primary influence on the landscape’s overall erosional efficiency.

**Fig. 17:**
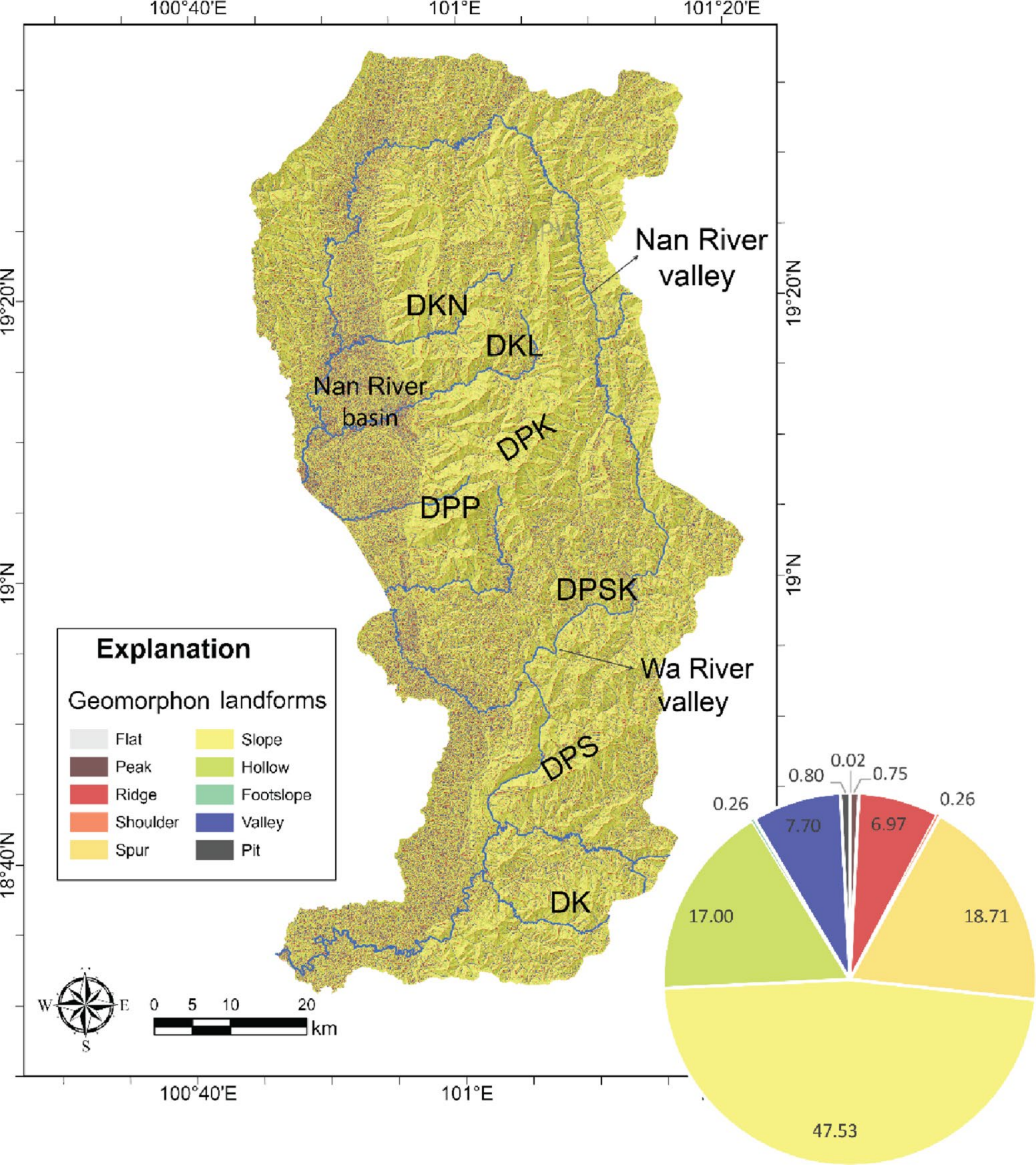
Geomorphon landform classification and percentage distribution of landform types in the Luang Prabang Range (LPR).

The spatial distribution of geomorphon landforms provides a structural framework that closely correlates with observed soil loss rates 158 (Fig. 17). Sloping terrain is the dominant landform type, covering almost half of the range, and reflecting the region’s high topographic complexity. The main geomorphic characteristics of the range are followed by spur-and-hollow landforms, which are intricately distributed across the central and eastern mountain ranges of DKN, DKL, and DPK. These landforms align with the high-elevation zones and rugged relief identified in the swath profiles. The prevalence of these sloping and convergent features likely governs surface runoff pathways and dictates the spatial patterns of soil erosion. Conversely, lower-relief valleys and ridge crests cover a smaller fraction of the landscape.

The synthesis of these datasets reveals a critical landform structure, and erosional processes directly drive landslide susceptibility. The rugged central and eastern mountains provide a complex structural framework that facilitates rapid surface runoff and deep soil incision, particularly where steep gradients intersect with vulnerable agricultural land. Consequently, these high-erosion, slope-dominated zones serve as primary predictors of landslide occurrences, as reflected in the high land-susceptibility percentages reported by advanced RF, XGBoost and CNN models. This interrelationship suggests that landslides in this region are not isolated events but are the catastrophic culmination of continuous soil degradation occurring across unstable geomorphic surfaces.

## Conclusion

This study presents a systematic evaluation of landslide susceptibility models in the Luang Prabang Range of Nan Province, Thailand, using statistical, machine learning (ML), and deep learning (DL) approaches within a unified, data-consistent framework. By applying all models with a comprehensive inventory of 1,752 historical landslide locations and identical 10 multi-source landslide conditioning factors, the results demonstrate that model performance is influenced not only by algorithm selection but also by data structure, sampling strategy, and parameter configuration.

Among the tested models, Random Forest (RF) exhibited the most stable and reliable performance, particularly under data-limited conditions. In contrast, deep learning models (CNN and LSTM) demonstrated strong predictive performance but were more sensitive to data availability and model configuration. These findings highlight that the commonly reported advanced models do not necessarily guarantee robust or transferable results in complex tropical mountainous environments. The key contribution derived from the study is the establishment of a controlled evaluation framework that enables a fair comparison of different modelling approaches. This framework provides critical insights into model robustness, uncertainty, and interpretability, addressing a major limitation in previous landslide susceptibility studies, where inconsistent data and validation strategies often obscure the actual model performance.

However, several limitations should be acknowledged. The use of environmental proxies instead of direct geotechnical measurements, reliance on long-term average rainfall rather than event-based triggers, and the absence of parameter optimization and ensemble modelling may influence the results. Future research should incorporate event-based rainfall analysis, climate change projections, and ensemble-based deep learning approaches to better capture the complex spatial–temporal dynamics of landslide processes. Overall, the study provides a robust baseline for landslide susceptibility mapping in data-constrained tropical regions and offers practical guidance for model selection. The findings emphasize that improving data quality, model interpretability, and evaluation consistency is as important as increasing model complexity for advancing reliable landslide hazard assessment.

## Data Availability

The data that support the findings of this study are available from the corresponding author upon reasonable request.
